# Electrical Microstimulation of the Pulvinar Biases Saccade Choices and Reaction Times in a Time-Dependent Manner

**DOI:** 10.1523/JNEUROSCI.1984-16.2016

**Published:** 2017-02-22

**Authors:** Adan-Ulises Dominguez-Vargas, Lukas Schneider, Melanie Wilke, Igor Kagan

**Affiliations:** ^1^Decision and Awareness Group, Cognitive Neuroscience Laboratory, German Primate Center, Leibniz Institute for Primate Research, Goettingen 37077, Germany,; ^2^Department of Cognitive Neurology, University of Goettingen, Goettingen 37075, Germany,; ^3^Deutsche Forschungsgemeinschaft Center for Nanoscale Microscopy and Molecular Physiology of the Brain, Göttingen 37075, Germany, and; ^4^Leibnitz Science Campus Primate Cognition, Goettingen 37077, Germany

**Keywords:** choice, decision-making, electrophysiology, microstimulation, pulvinar, saccades

## Abstract

The pulvinar complex is interconnected extensively with brain regions involved in spatial processing and eye movement control. Recent inactivation studies have shown that the dorsal pulvinar (dPul) plays a role in saccade target selection; however, it remains unknown whether it exerts effects on visual processing or at planning/execution stages. We used electrical microstimulation of the dPul while monkeys performed saccade tasks toward instructed and freely chosen targets. Timing of stimulation was varied, starting before, at, or after onset of target(s). Stimulation affected saccade properties and target selection in a time-dependent manner. Stimulation starting before but overlapping with target onset shortened saccadic reaction times (RTs) for ipsiversive (to the stimulation site) target locations, whereas stimulation starting at and after target onset caused systematic delays for both ipsiversive and contraversive locations. Similarly, stimulation starting before the onset of bilateral targets increased ipsiversive target choices, whereas stimulation after target onset increased contraversive choices. Properties of dPul neurons and stimulation effects were consistent with an overall contraversive drive, with varying outcomes contingent upon behavioral demands. RT and choice effects were largely congruent in the visually-guided task, but stimulation during memory-guided saccades, while influencing RTs and errors, did not affect choice behavior. Together, these results show that the dPul plays a primary role in action planning as opposed to visual processing, that it exerts its strongest influence on spatial choices when decision and action are temporally close, and that this choice effect can be dissociated from motor effects on saccade initiation and execution.

**SIGNIFICANCE STATEMENT** Despite a recent surge of interest, the core function of the pulvinar, the largest thalamic complex in primates, remains elusive. This understanding is crucial given the central role of the pulvinar in current theories of integrative brain functions supporting cognition and goal-directed behaviors, but electrophysiological and causal interference studies of dorsal pulvinar (dPul) are rare. Building on our previous studies that pharmacologically suppressed dPul activity for several hours, here we used transient electrical microstimulation at different periods while monkeys performed instructed and choice eye movement tasks, to determine time-specific contributions of pulvinar to saccade generation and decision making. We show that stimulation effects depend on timing and behavioral state and that effects on choices can be dissociated from motor effects.

## Introduction

The ability to decide flexibly between response options is a crucial attribute of adaptive behavior. One fundamental component of this process is the guidance of eye movements exploring spatial locations of potential interest. Representations of diverse variables contributing to saccadic decisions have been found in many cortical and subcortical brain regions (Andersen and Cui, 2009; Shadlen and Kiani, 2013). Based on the extensive anatomical connectivity to those regions, the thalamic pulvinar has been suggested as a hub for the coordination of movements for goal-directed visually-guided behavior (Grieve et al., 2000; Wilke et al., 2010). In primates, the pulvinar forms the largest thalamic complex and can be coarsely subdivided into ventral and dorsal aspects (Kaas and Lyon, 2007; Preuss, 2007). The ventral aspect is organized retinotopically and is connected with striate and extrastriate visual cortices. The dorsal aspect does not seem to contain an orderly retinotopic topography and is interconnected reciprocally with areas that combine spatial attention and eye movement functions, such as the parietal, superior temporal, posterior cingulate, and prefrontal cortices (Seltzer et al., 1996; Gutierrez et al., 2000; Jones, 2012). Both the ventral (vPul) and dorsal (dPul) pulvinar receive input from the superior colliculus (SC): the vPul from the upper and the dPul from the lower and intermediate layers of the SC (Stepniewska, 2004; Berman and Wurtz, 2011). Therefore, anatomical connectivity of the pulvinar suggests that it is involved in the selection and planning of eye movements and spatial attention.

Converging evidence is also provided by electrophysiological and lesion/inactivation studies. Visually responsive pulvinar neurons enhance firing for stimuli that are attended and/or are target of an upcoming saccade (Petersen et al., 1985; Robinson and Petersen, 1992; Bender and Youakim, 2001; Saalmann et al., 2012; Zhou et al., 2016). In addition, many pulvinar neurons exhibit saccade-related activity, including spatially specific enhancement or suppression associated with the onset of the visual target and/or onset or offset of the saccade (Petersen et al., 1985; Robinson et al., 1986, 1990; Berman and Wurtz, 2011). Studies of neural responses in eye movement tasks in the nonretinotopic, dorsal part of the pulvinar are particularly sparse, but suggest a diversity of saccade-related properties, with neurons exhibiting spatially untuned or direction-dependent perisaccadic and/or postsaccadic discharges (Robinson et al., 1986; Benevento and Port, 1995). Some medial dPul neurons have two peak responses, one closely following the onset of the visual target and the other triggered to the onset or offset of the saccade (Benevento and Port, 1995).

Pulvinar lesions in humans or monkeys do not result in primary visual or saccade generation deficits (Bender and Butter, 1987; Bender and Baizer, 1990; Van der Stigchel et al., 2010; Wilke et al., 2010, 2013), although a modest lesion-induced increase of contralesional saccade latencies has been reported (Rafal et al., 2004; Wilke et al., 2013). More pronounced are “higher-order” spatial attention and decision-making impairments (Robinson and Petersen, 1992; Saalmann and Kastner, 2011). Specifically, structural and reversible lesions in the vPul and/or dPul impair the ability to shift visual attention toward the contralesional hemifield and result in an ipsilesional spatial exploration and saccade choice bias (Rafal and Posner, 1987; Karnath et al., 2002; Arend et al., 2008; Snow et al., 2009; Wilke et al., 2010, 2013; Zhou et al., 2016). Although these lesion/inactivation studies provide strong evidence that normal pulvinar functioning is crucial for the selection of saccade goals in the presence of competing targets, they cannot resolve at which processing stage pulvinar exerts its effect on saccade behavior.

The aim of the current study was to investigate putative pulvinar-driven interactions between target selection and saccade generation in a temporally specific manner. To this end, we applied transient electrical microstimulation in the pulvinar while macaque monkeys performed visually- or memory-guided saccades to single (instructed) targets or chose between two targets in opposite hemifields. Crucially, we varied the timing of microstimulation, starting it before, at, or after onset of the saccade target(s). Our results demonstrate a temporal-specific impact of the pulvinar on spatial choices and saccade generation, further elucidating its involvement in goal-directed behaviors.

## Materials and Methods

### 

#### Procedures

All experimental procedures were conducted in accordance with the European Directive 2010/63/EU, the corresponding German law governing animal welfare, and German Primate Center institutional guidelines. The procedures were approved by the responsible government agency (LAVES, Oldenburg, Germany).

#### Animal preparation

Two adult male rhesus monkeys (*Macaca mulatta*), Monkey C and Monkey L, weighing 8 and 9 kg, respectively, were used. In an initial surgery, monkeys were implanted with an MRI-compatible polyetheretherketone (PEEK) head post embedded in a bone cement head cap (Palacos with gentamicin; BioMet) anchored by ceramic screws (Rogue Research) under general anesthesia and aseptic conditions. MR-visible markers were embedded in the head cap to aid the planning of the chamber in stereotaxic space (Monkey C, right hemisphere: center at 0.5 A/14.5 L mm, tilted −11 P/27 L degrees; Monkey L, right hemisphere: center at −3.12 P/20.2 L mm, tilted: −18 P/37 L degrees) with the MR-guided stereotaxic navigation software planner (Ohayon and Tsao, 2012). A separate surgery was performed to implant a PEEK MRI-compatible chamber (inside diameter 22 mm) allowing access to the right pulvinar. After confirming chamber positioning with a postsurgical MRI, a partial craniotomy was made inside the chamber. The exposed dura was covered with a silicone elastomer (Kwik-sil; World Precision Instruments) to reduce the granulation tissue growth and dura thickening.

#### MRI imaging

Monkeys were scanned in a 3 T MRI scanner (Magnetom TIM Trio; Siemens). Full-head T1-weighted scans (3D magnetization-prepared rapid gradient-echo, MPRAGE, 0.5 mm isometric) were acquired before and after chamber implantation in an awake (Monkey C) or anesthetized (Monkey L) state using either built-in gradient body transmit coil and custom single loop receive coil or custom single loop transmit and four-channel receive coil (Windmiller Kolster Scientific).

In addition to preimplantation and postimplantation scans, similar T1- and T2-weighted (rapid acquisition with relaxation enhancement, RARE, 0.25 mm in plane, 1 mm slice thickness) scans were acquired periodically during the course of experiments either in an awake (Monkey C) or sedated (Monkey L) state to confirm electrode positioning. T1- and T2-weighted scans were coregistered and transformed into “chamber normal” (aligned to the chamber vertical axis) and to AP–PC space for electrode targeting and visualization. These images were acquired with the chamber and the grid filled with gadolinium (Magnevist; Bayer)/saline solution (proportion 1:200) with tungsten rods inserted in predefined grid locations for alignment purposes.

#### Pulvinar targeting

The location of the electrode was estimated for every stimulation site based on anatomical MRI. Custom-made MR-compatible polyetherimide (Ultem) grids (0.8 mm hole spacing, 0.45 mm hole diameter) and custom-made plastic XYZ manipulator drives (design courtesy of Dr. Sebastian Moeller; Moeller et al., 2008) were used to position platinum-iridium electrodes (FHC, see detailed specs in the next section) in the corresponding grid hole and estimated depth. During the penetration, the electrode was protected by a custom-made MRI-compatible fused silica guide tube (320 μm inner diameter, 430 μm outer diameter; Polymicro Technologies) or a custom-made stainless steel guide tube (450 μm outer diameter, 27 gauge Spinocan; Braun Melsungen). A stopper (530 μm inner diameter, 665 μm outer diameter, 23 gauge MicroFil; World Precision Instruments) ensured that the guide tube only penetrated the dura and minimally the cortex below. Before penetration, the electrode tip was aligned to the guide tube tip and was held in place by a drop of melted petroleum jelly. The guide tube was filled with sterile silicone oil before electrode insertion to ensure smooth electrode travel and to prevent backflow of CSF.

There are multiple parcellation schemes available for the pulvinar (Stepniewska, 2004; Jones, 2012). Here, the pulvinar was divided into dPul and vPul aspects using the brachium of SC as a landmark, as has been done in several studies (Gutierrez et al., 2000; Wilke et al., 2010; Komura et al., 2013). The dPul includes medial pulvinar and dorsal part of lateral pulvinar (also denoted as PLdm, or Pdm in earlier studies; Robinson and Petersen, 1992), whereas vPul contains inferior pulvinar and ventral part of lateral pulvinar (also denoted as PLvl; Robinson et al., 1986; Kaas and Lyon, 2007). Because currently available online and downloadable atlases use the traditional scheme segregating medial (MPul), lateral (LPul,) and inferior (IPul) (and sometimes anterior/oral) nuclei (Rohlfing et al., 2012; Calabrese et al., 2015), we adopted this scheme for the localization of stimulation and recording sites.

**Figure 1.**
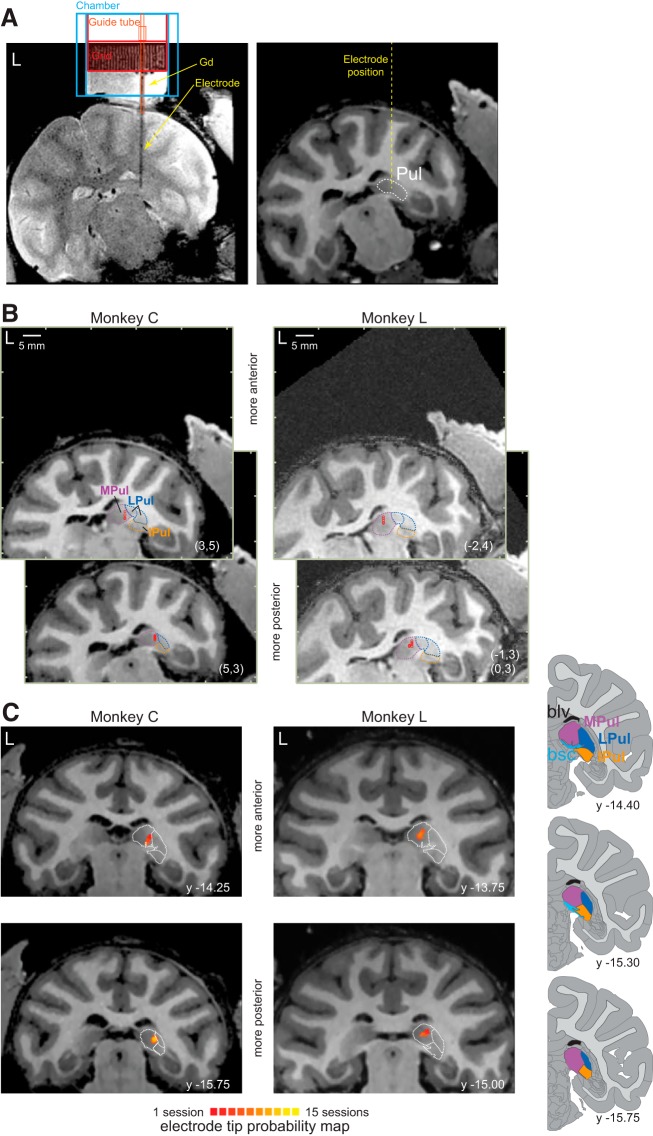
Localization of stimulation sites in the dPul. ***A***, Example scan of Monkey C with the stimulating electrode inserted in the pulvinar (grid location: *x* = 5, *y* = 3) with the chamber and the grid filled with the MRI contrast agent (gadolinium, Gd) (T2-weighted scan, left) and the corresponding section of the T1-weighted scan (right). ***B***, Electrode tip localization in individual stimulation sites (red circles) in chamber-normal coronal sections corresponding to specific grid locations (*x*, *y*; in parentheses) and depth. Pulvinar nuclei outlines (MPul, LPul, IPul) were adapted from the NeuroMaps atlas (Rohlfing et al., 2012), exported via the Scalable Brain Atlas (see https://scalablebrainatlas.incf.org/macaque/DB09 and https://scalablebrainatlas.incf.org/services/rgbslice.php (Bakker et al., 2015), and LPul was further subdivided to PLdm and PLvl. ***C***, Electrode tip localization probability maps in standard AC–PC space across all stimulation sites. The probability map was created by delineating a sphere of 0.5 mm radius around the tip in the chamber normal space for each stimulation session, transforming resulting volumes to AC–PC space and converting volumes of interest (VOIs) to a probability map using BrainVoyager VOI functions. Pulvinar nuclei outlines from the NeuroMaps atlas (dotted white) were scaled individually in the vertical and horizontal dimensions and overlaid on the corresponding anatomical sections. Right inset, Standard coronal sections (indicated by the *y*-coordinate) from the NeuroMaps atlas, going from anterior (top) to posterior (bottom). bsc, brachium of the SC; blv, body of the lateral ventricle.

As can be seen in Figure 1, the stimulation sites in the main experiment corresponded to the dPul, mostly to the MPul, but were also close to the dorsal aspect of the LPul. The brachium of the SC and other neighboring structures such as reticular thalamic nucleus and tail of the caudate nucleus were avoided.

#### Electrical microstimulation

An S88X dual output square pulse stimulator (Grass Products) triggered by a MATLAB-based task controller generated 200 ms trains of twin pulses at 300 Hz, which in turn triggered a constant current stimulus isolator A365 (World Precision Instruments) to produce 60 biphasic pulses. The current (100–300 μA, see below) was delivered using single monopolar electrodes (100 mm length platinum-iridium 125-μm-thick core, initial 2 cm glass-coated with an exposed tip of 40 μm, total thickness of 230 μm including polyamide tubing coating, UEPLEFSS (UEIK1; FHC); a return (reference) tungsten rod was placed in the chamber filled with saline. Voltage drop across a 10 kΩ resistor in series with the electrode was monitored using a four-channel 1GS/s Tektronix TDS2004C oscilloscope.

The manufacturer-specified impedance of electrodes was 300–500 kΩ; the initial impedance measured at 1000 Hz before the experiment was 360–1300 kΩ. Because the impedance dropped dramatically after a few stimulation trains were applied, before each session, 10 trains were delivered to the electrode immersed in saline using 300 μA current to bring the electrode impedance to a more stable regime. After this procedure, the impedance ranged from 19 to 200 kΩ for electrodes used in Monkey C and from 11 to 100 kΩ in electrodes used in Monkey L (see Table 1).

#### Electrophysiological recordings

In 19 sessions in Monkey C and 28 sessions in Monkey L right dPul neuronal activity was recorded with up to three individually movable single platinum-tungsten (95–5%) quartz glass-insulated electrodes with impedance ranging from 1 to 1.9 MΩ for Monkey C and from 1.3 to 3.5 MΩ for Monkey L using a chamber-mounted five-channel Mini Matrix microdrive (Thomas Recording). The recording target locations were estimated similarly to the stimulation sessions using the same grids. Similar to microstimulation experiments, single custom-made stainless steel guide tubes (27 gauge) filled with the silicone oil (Thomas Recording) with a Spinocan funnel attached to the drive nozzle were used to protect electrodes during dura penetration. A reference tungsten rod or a silver wire were placed in the chamber filled with saline and were connected to the chassis of the drive. Neuronal signals were amplified (20× headstage, Thomas Recording; 5×, 128 or 32 channel PZ2 preamplifier, Tucker-Davis Technologies), digitized at 24 kHz and 16 bit resolution, and sent via fiber optics to an RZ2 BioAmp Processor (Tucker-Davis Technologies) for online filtering, display, and storage on a hard drive together with behavioral and timing data streams.

#### Behavioral tasks

Monkeys sat in a dark room in custom-made primate chairs with their heads restrained 30 cm away from a 27-inch LED display (60 Hz refresh rate, model HN274H; Acer). The gaze position of the right eye was monitored at 220 Hz using a MCU02 ViewPoint infrared eyetracker (Arrington Research). Monkey face and body were monitored with infrared cameras to ensure that microstimulation did not elicit abrupt movements or signs of discomfort. All stimulus presentation and behavioral control tasks were programmed in MATLAB (The MathWorks) and the Psychophysics Toolbox (Brainard, 1997).

##### Fixation task and evoked saccades.

**Figure 2.**
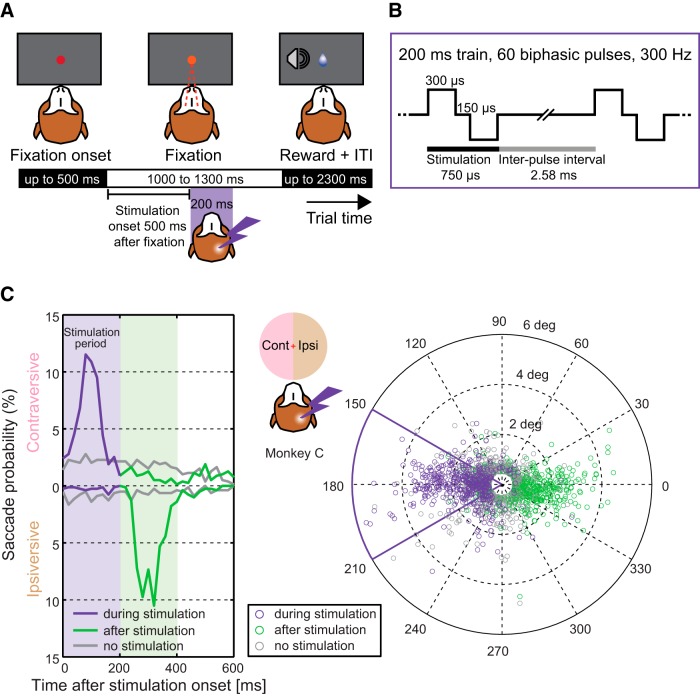
Fixation task and characterization of evoked saccades. ***A***, Task layout. Monkeys fixated a central spot for a variable time to receive liquid reward. In half of the trials, we applied a train of biphasic electric pulses to characterize potential evoked saccades. ***B***, Stimulation parameters. Each 200 ms stimulation train consisted of 60 biphasic pulses applied at 300 Hz (two pulses are shown). Each biphasic pulse started with a 300 μs positive phase, followed by 150 μs interphase interval and a 300 μs negative phase. There was a 2.58 ms interval between pulses. ***C***, Saccade probability distribution as a function of time during and after stimulation (left) and corresponding saccade end points (right). Saccades that started during the stimulation period are shown in purple, saccades that started in the 200 ms window after stimulation in green, and saccades in trials without stimulation in gray. Plotted data are from 1580 fixation trials in Monkey C (15 stimulation sites). Only saccades with amplitudes >0.5° were included in this analysis. Left, Time axis relative to stimulation onset or a corresponding time in control trials. The probability of contraversive or ipsiversive saccades is shown as upward and downward histograms, respectively (bin 20 ms). Right, Saccade direction and amplitude in the fixation task. End points are shown relative to each saccade starting position. We defined evoked saccades as saccades during the stimulation period that were followed by a saccade to the opposite side, returning to the fixation spot. The evoked saccades occurred mainly along the horizontal axis to the contraversive side: 83% were contained within a 30° angle below and above the horizontal axis (solid purple sector outline).

In each microstimulation session before the main visually-guided saccade task (see below), five to six blocks of 20 fixation trials (see Fig. 2*A*) were performed to determine the presence/absence of evoked saccades as a consequence of electrical stimulation. A dim red spot of 1° diameter (luminance 9.4 cd/m^2^) appeared in the center of the monitor (0.16 cd/m^2^). Once the monkeys directed their gaze into a 5° radius window surrounding the fixation spot, it became brighter (33 cd/m^2^) to signal fixation acquisition. Monkeys were required to maintain their gaze position for a randomized period ranging from 1000 to 1300 ms to complete a trial successfully before receiving liquid reward. The intertrial interval (ITI) was 1000 to 2000 ms. In half of the trials of each block, a stimulation was applied starting 500 ms after fixation was acquired. Each session started with a block of 100 μA and, in each subsequent block, current was increased by 50 μA until the 300 μA limit was reached. This range of currents was selected to match related ongoing fMRI/microstimulation experiments in our laboratory. The presence or absence of evoked saccades in a given block was assessed by online monitoring and all sessions were characterized offline (see below). If no evoked saccades were observed with any of the currents, the following tasks were performed with 250 μA; otherwise, the current was set to 50 μA below the lowest intensity that evoked saccades. If all current strengths evoked saccades and if, according to our MRI-based estimates, after moving the electrode, it still would be within 1 mm of the targeted pulvinar nucleus borders, the electrode was moved by 0.5 or 1 mm up or down and five blocks of fixation trials were run again (10 of 15 sessions in Monkey C, 0 of 15 in Monkey L). Alternatively, the highest current that did not evoke more saccades than the 100 μA current was used (two of 15 sessions in Monkey C, zero of 15 in Monkey L). In five of 15 sessions in Monkey C, the electrode was moved and the current was lowered below 250 μA even after moving the electrode.

The offline analysis confirmed online observations. When data from all fixation trials were combined, Monkey L did not show any difference in amount of saccades during stimulation compared with the same period during control trials (4% and 4%, respectively; 2% contraversive and 2% ipsiversive in each case). Monkey C, which incidentally had more frequent “fixational” saccades within the 5° radius fixation window even in control trials, exhibited predominantly contraversive saccades during the stimulation period (Fig. 2*C*; 60% of stimulation trials, 57% contraversive, 3% ipsiversive; 32% of control trials in the corresponding period, 22% contraversive, 10% ipsiversive). Contraversive saccades were typically followed by ipsiversive saccades (69%) within up to 200 ms after stimulation offset. Because the monkey was required to maintain fixation during the stimulation and these ipsiversive saccades were directed back to the fixation spot, we call them “return” saccades (82% of return saccades were preceded by contraversive ones). For further analysis, we classified as evoked saccades only contraversive saccades during the stimulation period that were followed by return saccades. We normalized the probabilities of evoking saccades per current strength for each site by subtracting the mean values for each site and found that the normalized probability of evoking saccades correlated with the current strength (Spearman's *r* = 0.38, *p* < 0.001). A similar analysis for evoked saccade amplitudes also showed a positive correlation with the current strength (Spearman's *r* = 0.31, *p* = 0.009). Across all sites that were later used in the main experiment and across all tested currents, the probability of evoking saccades in Monkey C was 39% (40.5% for the currents selected for the main experiment; see Table 1; for comparison, only 2% of control trials would have been classified as “evoked” using the above approach). The amplitude of evoked saccades was 1.51 ± 0.16° (mean ± SE) across sites (1.9 ± 1°, mean ± SD across trials), with a latency of 95 ± 39 ms after stimulation onset. Similar effects (increased probability of contraversive movements during stimulation period) were observed in several sessions in which we delivered the stimulation during free-gaze exploration (Goldberg et al., 1986; Watanabe and Munoz, 2013), with the exception of not observing ipsiversive return saccades. Our observations are consistent with lateral posterior nucleus/pulvinar microstimulation studies in cats, which reported either absence of evoked saccades (Maldonado et al., 1980) or contraversive saccades with current strengths between 50 and 300 μA (Crommelinck et al., 1977).

Although evoked saccades were present only in some sites, with <50% probability and only in one monkey, we will briefly address relevant methodological considerations. Due to small amplitudes, the visual and positional consequences of these saccades are expected to be relatively minor (Carello and Krauzlis, 2004), although we cannot exclude the possibility of perceptual/attentional effects similar to consequences caused by fixational saccades (Hafed et al., 2015). Given the 24° target eccentricity in the saccade tasks (see below), these displacements were not enough to land the gaze within the target window and did not seem to affect the ensuing choice. For example, during choice trials, when a small contraversive shift was apparent in the online display (and later during inspection of trial eye position traces), the monkey would often go on to select the ipsiversive target even though his gaze was already closer to the contraversive target.

At those sites where microstimulation evoked small saccades, required current strength was considerably higher than reported for SC, caudate nucleus or frontal eye fields (FEFs) (Robinson and Fuchs, 1969; Tehovnik et al., 1999; Yamamoto et al., 2012). Instead, the range of evoked saccade thresholds between 100 and 300 μA was more similar to required currents in visuomotor regions such as posterior parietal cortex (Shibutani et al., 1984; Thier and Andersen, 1996) and dorsomedial frontal cortex (Tehovnik et al., 1999).

##### Visually-guided saccade task.

**Figure 3.**
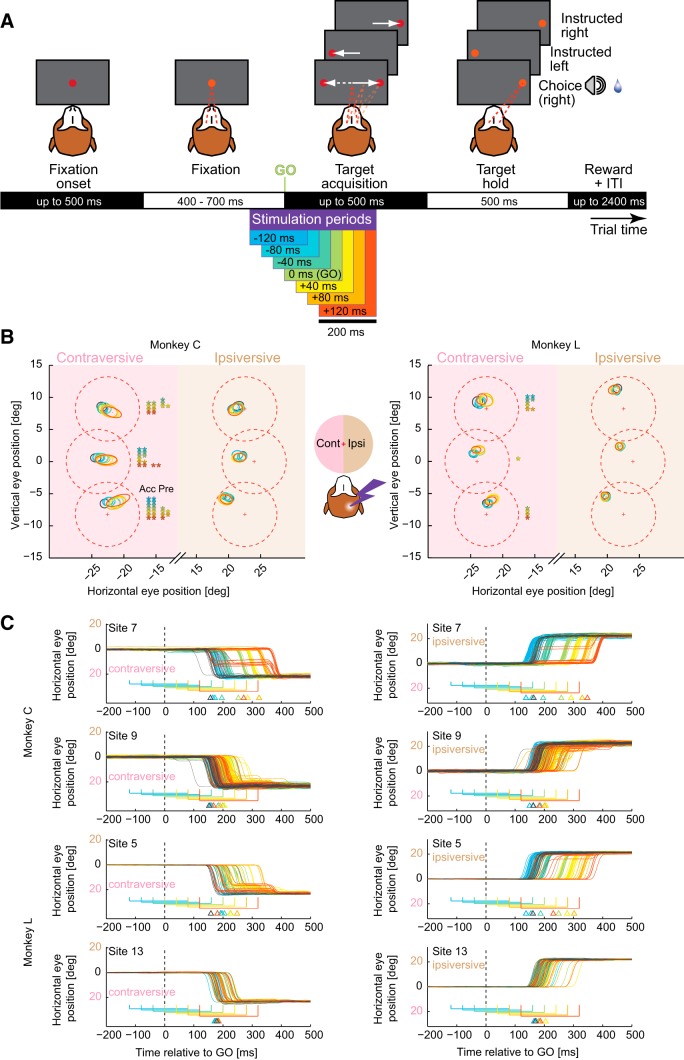
Visually-guided saccade task. ***A***, Task layout. Stimulation was delivered in one of seven different periods: starting before the target(s) onset (−120 ms, −80 ms, or −40 ms), at the Go signal, or after the Go signal (+40 ms, +80 ms, or +120 ms). Trials without stimulation were interleaved as a control. The color code for each stimulation period is same for all following figures. ***B***, Saccade accuracy and end point scatter. Dashed red circles represent the allowed 5° radius of target acquisition window; crosses represent the targets center. Half-axes of colored ellipses (control: gray) represent the means, across sessions, of SDs of radial and angular coordinates of end points; ellipse centers are means of mean end points across sessions. Both monkeys showed reduced accuracy (“Acc,” the distance from the target center to the mean end point) and increased end point scatter in the contraversive side of space (precision, “Pre,” the radial component corresponding to ellipse major axis). The asterisks denote significant effects Acc and Pre separately for each target position (*p < 0.05, **p < 0.01, Friedman with *post hoc* Wilcoxon signed-rank test, Bonferroni corrected). These accuracy and precision effects did not impair monkeys' ability to acquire any of the targets (Fisher's exact test within each session, Bonferroni corrected, *p* > 0.05; Table 2). ***C***, Horizontal eye position traces for the different stimulation periods in two example sessions in Monkey C (top two rows) and Monkey L (bottom two rows) for one pair of contraversive (left column) and ipsiversive (right column) targets, successful trials, aligned to the Go signal (0 ms, dotted vertical lines). The color of the traces represents corresponding stimulation periods, which are also shown as brackets below the traces (control: gray). The triangles below denote mean RT for each period.

A trial started with the onset of the fixation spot. After the monkey acquired and held fixation within a 5° radius for a randomized period ranging from 400 to 700 ms, the fixation spot (1° diameter) was extinguished and either one target (instructed trials) or two targets (choice trials) appeared simultaneously (see Fig. 3*A*). This time point will be referred to as the “Go signal.” Targets (1° diameter) were presented in the left and/or right side(s) of the fixation spot, at 24° eccentricity, with 3 potential angles relative to the horizontal axis: 0°, 20°, or −20° (0°, 8.2°, and −8.2° vertical eccentricity). Monkeys had to make a saccade within 500 ms and keep their gaze position for 500 ms inside a 5° radius window surrounding the target to complete a trial successfully and obtain a liquid reward after a delay of 200 ms. In choice trials, monkeys were allowed to choose one of the targets freely; both choice targets were always presented at the same height and provided equal reward. The ITI for both successful and unsuccessful trials was 1000 or 2000 ms. In seven of eight trials, a 200 ms stimulation train was applied at one of seven different periods in both instructed and choice trials. The trains started either before the Go signal (−120 ms, −80 ms, or −40 ms; early stimulation periods), simultaneously with the Go signal, or after the Go signal (+40 ms, +80 ms, or +120 ms; late stimulation periods). Note that because the train duration was 200 ms, stimulation always ended after the Go signal. All trial types, target locations, and stimulation conditions were pseudorandomized. A minimum of 15 instructed trials per stimulation period and per hemifield were collected in each session (except in one session, where there was a minimum of 13 trials for the left hemifield and a session with 14 trials for the right hemifield).

##### Memory-guided saccade task.

Similarly to the visually-guided saccade task, monkeys had to acquire and hold fixation for 400–700 ms. Next, one or two peripheral cues were displayed for 280 ms at the location(s) signaling the upcoming saccade target(s). These cues had the same spatial characteristics as the targets in the visually-guided task. Monkeys were required to maintain fixation throughout the cue period and also throughout the subsequent memory period (ranging from 200 to 400 ms), after which the central fixation spot disappeared (Go signal), allowing monkeys to saccade to the instructed location or make a decision to go to one of the two cued locations. After the saccade to and fixation of the remembered target location for 100 to 200 ms, the target became visible and, after an additional 500 ms of peripheral fixation, the trial was completed. We applied stimulation in four of five trials in one of four periods starting before or after the cue onset (−80 ms, +80 ms) or before or after the Go signal (−80 ms, +80 ms).

##### Target selection equalization.

During training, we consistently observed a strong selection bias to the right side of space in choice trials in both monkeys. This bias was potentially due to the fact that both monkeys were initially trained to perform reaches with their preferred right arm in the context of another experiment, in which they might have developed a strong rightward bias. To be able to assess potential target selection changes in both directions due to stimulation, we used a method similar to that used by Scherberger et al. (2003) to equalize the control target selection by shifting the entire stimulus array horizontally toward the preferred right hemifield without modifying the 24° eccentricity from the fixation spot to the targets. The mean shift across visually-guided task sessions with stimulation in dPul was 3.2 ± 0.8° for Monkey C and 4.4 ± 0.6° for Monkey L to the right (mean ± SE; see Table 1). These shifts resulted in the 44 ± 3% and 40 ± 6% leftward selection in prestimulation runs that were used for the equalization procedure (Monkeys C and L, respectively, mean ± SE; see Table 1). However, during the actual stimulation experiment, the leftward (contraversive) selection dropped to 29 ± 5% and 26 ± 5% in nonstimulation trials (Monkeys C and L, respectively). The same target positions were used for instructed trials.

##### Summary of the course of a session.

After advancing the electrode to the desired location, the fixation task (with the fixation spot always in the center of the screen) was used to test for evoked saccades. This procedure defined the final electrode depth and the current strength. Next, a visually-guided saccade task was performed without stimulation and the stimulus array was shifted to find a regime in which the left and right target selection was approximately equalized (see “Target selection equalization” section above). After that, the control and stimulation data for the main visually-guided task was collected. For sessions in which monkeys also performed the memory-guided saccade task, target selection was equalized independently for the memory task because target preference differed between the two tasks.

#### Data analysis

##### Saccade definitions.

Saccade velocity was calculated sample by sample as the square root of the sum of squared interpolated (220 Hz to 1 kHz), smoothed (12 ms moving average rectangular window) horizontal and vertical eye position traces, and then smoothed again (12 ms moving average rectangular window). Saccade onset was defined as eye position change that exceeded a starting velocity threshold and the saccade offset as reaching an ending velocity threshold. For the fixation task, the starting and ending thresholds were 30°/s and 15°/s, respectively. For visually-guided and memory-guided saccades, a starting velocity of 300°/s and ending velocity of 50°/s were used. For retrieving the saccade directions in error trials, the starting threshold was lowered to 150°/s because eye position was not recorded after fixation breaks, so in some cases, the recorded velocity did not reach a high enough value before the trial and the recording were aborted. Saccade end point was defined as the eye position when the saccade velocity reached the ending threshold. In cases when several consecutive eye movements in the time interval from the Go signal until the target acquisition fitted the above criteria (e.g., due to interrupted saccades; see Results), the first saccade was selected for the reaction time (RT) analysis and the last one for the end point accuracy/precision analysis.

##### Statistical analysis of behavioral data.

All data analysis was performed using MATLAB R2012b. To test for changes in target selection preference within each session and the hit rates, Fisher's exact test was used. For all comparisons between two conditions across sessions, nonparametric tests were used. Whenever possible (i.e., same experimental conditions/outcomes present in all stimulation periods and in all sessions), paired Friedman test with *post hoc* Wilcoxon signed-rank tests were used. Otherwise, Kruskal–Wallis test with *post hoc* Mann–Whitney *U* tests were used. Because the effects of multiple stimulation periods were tested against the control condition, for all *post hoc* tests and for Fisher's exact tests, the Bonferroni method was used to correct for multiple comparisons. To test for the relationship between two variables across sessions or across stimulation periods, Spearman's correlation coefficients were used. Statistical significance was reported at *p* < 0.05 (*) and *p* < 0.01 (**). Specific statistical tests are listed for each individual analysis. In the figures and in the text, SD was used when averaging across trials and SE when averaging across sessions.

##### Analysis of neuronal activity.

In the data from both monkeys, 230 single and multiunits for the visually-guided saccade task (140 Monkey C, 90 Monkey L) and 365 units for the memory-guided saccade task (251 Monkey C, 114 Monkey L) fulfilled analysis selection criteria (at least 50 spikes during the task periods; at least 60 instructed trials; typically 120 instructed trials, 10 instructed trials for each of the 12 targets). For recordings, the fixation hold period was 500 ms, the memory period 1000 ms, and the ITI period 1000 ms; other parameters were same as in the stimulation runs. Target eccentricities were 12° and 24°, arranged along the horizontal axis or at a ±20° angle from the horizontal axis. Spike sorting was done using Offline Sorter versions 4.0.0 and 2.8.8 (Plexon) for Monkeys C and L, respectively, using either a waveform template algorithm or a principle component analysis with k-means clustering algorithm.

For each trial and each epoch of interest, firing rates were computed by counting the spikes within the epoch and dividing the count by the epoch duration. The epochs analyzed in the visually-guided saccade task were “ITI” (400 to 100 ms before the onset of the central fixation spot), “fixation acquisition” (50 to 150 ms after acquiring central fixation), “fixation hold” (last 300 ms of central fixation), “target onset” (50 to 150 ms after target onset), “presaccadic” (100 to 10 ms before saccade onset), “perisaccadic” (10 ms before to 50 ms after saccade onset), “target acquisition” (50 to 120 ms after acquiring target fixation), and “target hold” (last 300 ms of fixating the peripheral target). For the memory-guided saccade task, “cue onset” (50–150 ms after onset of the cue) replaced the “target onset” and both will be referred to as “stimulus onset”; “target hold invisible” (first 100 ms of fixating the invisible peripheral target) replaced “target acquisition.” Two additional epochs were also analyzed: “early memory” (first 200 ms of the memory period) and “late memory” (last 300 ms of the memory period).

For population analysis, data from six left and six right hemifield targets were combined. For each unit, a two-way ANOVA was performed across all firing rates in each of the respective epochs from successful instructed trials (same criteria as in “Behavioral tasks” section) using hemifield of the target position and epoch as factors for determining a main effect of epoch, hemifield, and interaction between the two. Spatial tuning in each epoch was determined by unpaired *t* tests comparing firing rates in ipsilateral trials with firing rates in contralateral trials. The hemifield with the higher firing rate was marked if there was a significant difference. This analysis was performed only on units that showed either a main effect of hemifield or a hemifield × epoch interaction.

Enhancement or suppression of neuronal activity (relative to fixation baseline, “fixation hold” epoch) in each subsequent epoch was defined by paired *t* tests comparing firing rates for ipsilateral and contralateral trials independently. This analysis was only performed on units that showed either a main effect of epoch or hemifield × epoch interaction. Enhancement or suppression was reported if either ipsilateral, contralateral, or both types of trials showed a significant difference from fixation baseline. In rare cases in which one hemifield would show a significant enhancement while the other hemifield showed suppression, the unit was reported to have bidirectional response (example unit counts, memory-guided task, Monkey C/Monkey L: cue 2/1, perisaccade 2/0, target hold: 6/1; visually-guided task, Monkey C/Monkey L: target onset: 2/0, perisaccade: 3/2, target hold: 4/4).

For response field (RF) estimation, an independent one-way ANOVA was performed on firing rates during the stimulus onset epoch for each unit to determine the effect of target position. For defining a hemifield preference, the hemifield with the higher firing rate was marked if there was an effect of target position. For all units that showed an effect of target position, response modulation depth for each target position was calculated by averaging firing rates across trials, subtracting the lowest average firing rate across positions, and converting to percentage of maximal modulation depth. To estimate the center and size of the RFs, a 2D Gaussian was fitted to the modulation depth pattern. Six fitting parameters were determined using an iterative least-squares method (400 iterations), allowing elliptic RFs with peaks at the center. The size of the RF was defined by 2 SDs in each direction (semi-minor and semi-major axes). The fitting parameters were as follows: (1) the modulation depth in the center of the RF, (2) horizontal and (3) vertical location of the RF center, (4 and 5) ellipse major axis defined by 4 SDs and minor axis by aspect ratio, and (6) an angle of ellipse rotation. Importantly, the RF center was always kept within the dimensions of the target array (−24° to +24° horizontally and −8.2° to +8.2° vertically). The amplitude was bounded by 50% and 150% of the original modulation depth, ellipse axes were bounded by 12° (maximum horizontal distance between targets) and 48° (target array extent), and maximum aspect ratio 4:1. Maximum modulation depth, the average of modulation-depth-weighted target positions (RF “center of mass”), intermediate major/minor axes (30°/15°), and a rotation of 0° were used as starting values for the fits.

An averaged radius (*r*) approximating the RF size was calculated by taking the square root of the product of the two axes of the elliptic RF. This way, *r*^2^*π always matches the area covered by the elliptic RF. RF size is reported as the diameter of the RF, 2*r.

For target hold and “stimulus onset” epochs (cue onset for memory-guide saccades and target onset for visually-guided saccades) contralateral tuning indexes (CI) for each unit were calculated as follows: CI = (FR_contra_ − FR_ipsi_)/(FR_contra_ + FR_ipsi_), where FR_contra_ and FR_ipsi_ are the average firing rate for all trials with targets in the contralateral and ipsilateral hemifield, respectively. Positive indexes indicate contralateral preference and negative indexes indicate ipsilateral preference.

To calculate population peristimulus time histograms, spike density functions of each trial, derived by convolution of the discrete spike arrival times with a Gaussian kernel (SD 20 ms) were baseline corrected by subtracting the average ongoing firing rate in the late period of the ITI that immediately preceded the trial start (fixation spot onset). Average responses for each unit were then derived by averaging the baseline-corrected spike density for each unit across all trials for the respective condition. Mean and SE of these baseline-corrected and averaged spike densities across units of a given subpopulation were calculated to display population responses. For better visualization of target position-dependent population cue response, instead of subtracting a baseline, the response was normalized by dividing each unit's response in all conditions by the same factor. That factor was defined as the peak firing rate during the cue onset epoch calculated across all trials (regardless of target position) in the preferred hemifield.

## Results

Using an MRI-guided approach (see Materials and Methods), we stimulated the right dPul (Fig. 1, Table 1) and control sites in the vPul (see later section) in two monkeys performing three oculomotor tasks: fixation, visually-guided saccades, and memory-guided saccades (to instructed or chosen locations). The fixation task (Fig. 2*A*) was used to test for occurrence of evoked saccades and to characterize them if present. Monkey L did not exhibit evoked saccades in the regime tested; Monkey C showed predominantly small (<2°) contraversive saccades at 95 ± 39 ms after the stimulation onset (Fig. 2*C*; Materials and Methods). The visually-guided task was used in the main experiment and the memory-guided task was used as a control for dissociating the cue processing, motor planning, and execution phases (see below). Both saccade tasks included 50% single-target-instructed trials and 50% choice trials between two equally rewarded targets located equidistantly from the central fixation spot at the same height.

**Table 1.** Summary of 56 stimulation datasets

Monkey-site-task-session no.	Trials (*n*)	Offset (°)	Contraversive choice in prestimulation runs (%)	Current (μA)	Impedance (kΩ)	
					Before conditioning	After conditioning
L-dPul-V-1	566	3	80	250	-	100
L-dPul-V-2	960	3	31	250	80	60
L-dPul-V-3	546	3	22	250	80	60
L-dPul-V-4	696	6	22	250	-	50
L-dPul-V-5	625	3, 6	20	250	-	50
L-dPul-V-6	1895	0, 3	65	250	250	50
L-dPul-V-7	960	0	21	250	700*	29
L-dPul-V-8	960	5	24	250	19	14
L-dPul-V-9	960	5	21	250	160	32
L-dPul-V-10	960	8	52	250	200	60
L-dPul-V-11	960	5	45	250	100	40
L-dPul-V-12	960	5	34	250	180	25
L-dPul-V-13	480	3	41	250	65	24
L-dPul-V-14	480	7	84	250	60	28
L-dPul-V-15	480	7	36	250	600*	22
C-dPul-V-1	818	0	49	250	105	60
C-dPul-V-2	1248	5	31	100, 200	45	32
C-dPul-V-3	1276	5	27	200, 250	170	35
C-dPul-V-4	960	5	42	200	22	22
C-dPul-V-5	1440	5	25	150, 250	360*	28
C-dPul-V-6	1158	5	31	150, 250	23	23
C-dPul-V-7	960	0	62	250	1100*	33
C-dPul-V-8	960	5	49	150	1100*	33
C-dPul-V-9	1920	0, 5	38	250	1300*	65
C-dPul-V-10	960	2	43	250	390*	32
C-dPul-V-11	960	0	37	250	65	28
C-dPul-V-12	480	−2	47	250	75	20
C-dPul-V-13	480	0	51	150	600*	19.5
C-dPul-V-14	480	10	64	250	170	-
C-dPul-V-15	864	6	61	200	440*	38
L-dPul-M-1	600	−5	22	250	60	28
L-dPul-M-2	600	5	54	250	600*	22
C-dPul-M-1	600	3	89	150	600*	19.5
C-dPul-M-2	600	6	53	200	440*	38
C-dPul-M-3	240	10	93	250	170	-
L-vsPul-V-1	960	0	36	250	700*	25
L-vsPul-V-2	960	2	19	200	850*	20
L-vsPul-V-3	960	3	19	250	33	25
C-vsPul-V-1	960	2	37	250	600*	200
C-vsPul-V-2	960	5	34	250	37	35
C-vsPul-V-3	960	0	71	150	41	31
L-vmPul-V-1	960	4	11	250	140	29
L-vmPul-V-2	1920	3	22	150, 200	850*	12
L-vmPul-V-3	960	2	19	250	25	20
L-vmPul-V-4	960	3	33	250	40	21
L-vmPul-V-5	960	3	19	250	19	11
C-vmPul-V-1	960	0	66	250	41	31
C-vmPul-V-2	960	0	50	250	600*	60
L-vdPul-V-1	960	3	32	200	40	21
L-vdPul-V-2	960	3	19	250	19	11
L-vdPul-V-3	960	3	19	250	33	25
C-vdPul-V-1	960	2	46	250	28	19
C-vdPul-V-2	960	0	44	250	37	35
C-vdPul-V-3	960	0	33	250	41	31
C-vdPul-V-4	960	0	59	250	600*	60
C-vdPul-V-5	960	0	46	200	200	19

Offset is the horizontal shift of the entire stimulus array (fixation point and targets) from the center of the screen; positive values indicate shift to the right. Current is the current strength used in the visually-guided or memory-guided task. Impedance before conditioning refers to the electrode impedance before applying 10 200 ms 300 μA trains outside of the brain. First-time-use electrodes (out of the box) are marked with asterisks. Impedance after conditioning refers to resulting impedance after applying conditioning stimulation trains.

C, Monkey C, L, Monkey L; vs, ventral shallow; vm, ventral medium; vd, ventral deep; V, visually-guided task; M, memory-guided task.

### Visually-guided task: time-dependent RT facilitation and delay

In the visually-guided saccade task (Materials and Methods), in stimulation trials, a 200 ms train was delivered at different periods relative to the target(s) onset and synchronous fixation spot offset, referred to as “Go” signal: before “Go” (early periods, blue–cyan colors), at “Go,” or after “Go” (late periods, green–orange colors) (Fig. 3*A*). All trial conditions, instructed/choice, contraversive/ipsiversive with respect to the stimulated right hemisphere (left hemifield/contraversive, right hemifield/ipsiversive), stimulation/no stimulation, and different stimulation periods, were interleaved randomly.

In instructed trials (single targets), the stimulation did not affect the hit rate (the fraction of successfully completed trials), which remained consistently high (Table 2). However, stimulation caused mildly hypometric saccades for contraversive locations: saccades were still initiated in the correct direction but often undershot, resulting in reduced end point accuracy and increased scatter along the saccade trajectory axis, predominantly in late stimulation periods during premovement and movement phases (Fig. 3*B*), similar to findings in SC (Schlag et al., 1989) and pre-SMA (Isoda, 2005).

**Table 2.** Hit rates in the visually-guided saccade task, instructed trials, dorsal pulvinar stimulation (mean ± SE across sessions)

Stimulation period onset	Contraversive hit rate (%)			Ipsiversive hit rate (%)		
	Both monkeys	Monkey C	Monkey L	Both monkeys	Monkey C	Monkey L
Control (no stimulation)	99 ± 0	99 ± 1	100 ± 0	98 ± 1	100 ± 0	97 ± 1
−120 ms to Go	100 ± 0	99 ± 1	100 ± 0	98 ± 1	98 ± 1	98 ± 1
−80 ms to Go	99 ± 0	99 ± 1	100 ± 0	99 ± 0	99 ± 0	99 ± 1
−40 ms to Go	99 ± 0	99 ± 1	100 ± 0	99 ± 0	100 ± 0	99 ± 0
Go (target onset)	98 ± 1	98 ± 1	99 ± 0	97 ± 1	98 ± 1	96 ± 1
+40 ms from Go	97 ± 1	96 ± 1	99 ± 1	98 ± 0	98 ± 1	98 ± 1
+80 ms from Go	98 ± 1	97 ± 2	99 ± 1	97 ± 1	98 ± 1	97 ± 1
+120 ms from Go	97 ± 1	95 ± 2	100 ± 0	96 ± 1	97 ± 1	95 ± 2

The main effect of the stimulation on saccade performance consisted of changes in RTs. To illustrate this, we plotted the horizontal eye position as a function of time in the control (no stimulation) trials and in the different stimulation periods for two target positions in two example sessions in each monkey (Fig. 3*C*). Three apparent effects of stimulation can be gleaned from these plots: (1) RT delay in most periods with saccade onsets either stereotypically deferred until after the stimulation offset (Fig. 3*C*, top example in each monkey) or delayed yet initiated during the stimulation (Fig. 3*C*, bottom example in each monkey) for saccades in both directions; (2) RT facilitation for ipsiversive saccades in early stimulation periods; and (3) occurrence of interrupted saccades (movement stopping in the mid-fly) in the late stimulation periods, especially for the contraversive targets.

**Figure 4.**
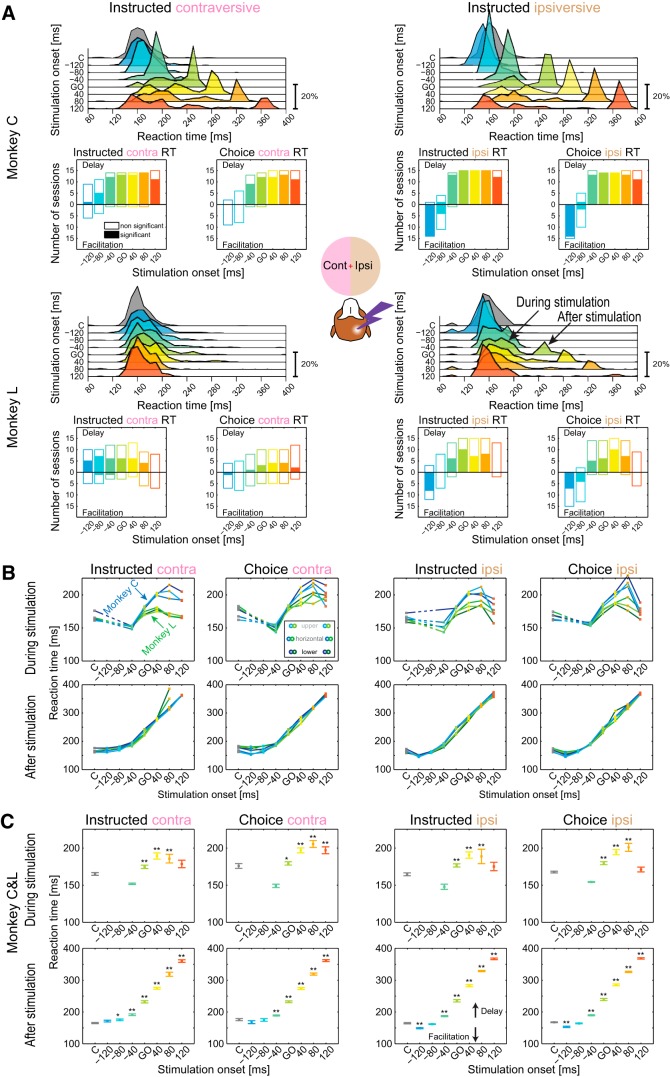
Effect of stimulation on RTs in the visually-guided saccade task. ***A***, Stimulation effects on RTs for contraversive (left) and ipsiversive (right) saccades for Monkey C and Monkey L, respectively. Top, RT histograms (10 ms bin, normalized to 100% per condition) show data across all trials for each stimulation period. For stimulation onsets at or after the Go signal, the saccade initiation was delayed compared with control (gray). The saccade initiation was either delayed or arrested until the end of the stimulation period, resulting in a bimodal RT distribution for the late stimulation periods. In ipsiversive trials for the earliest stimulation period, −120 ms to the Go signal, there was a facilitatory effect on saccade onsets. Bottom, Session by session directionality and significance of RT effect. For each stimulation period, each session RT that differed from control trials is shown as either a positive or negative bin, representing either delay or facilitation. Filled bins represent sessions in which the change from control was significant (Friedman followed by Mann–Whitney *U* test, Bonferroni corrected). It should be noted that, for ipsiversive saccades, the facilitation effects at the two early stimulation periods (−120 and −80) were consistently present in both monkeys in both choice and instructed trials. ***B***, Summary of RT effects for contraversive (left) and ipsiversive (right) saccades, separated by monkey (blue and green traces are for Monkey C and Monkey L, respectively) and by vertical target position (light-, medium-, and dark-shaded traces denote upper, horizontal, and lower positions, see inset). Plots show saccades that started either during stimulation period (top row) or after stimulation offset (bottom row). Dashed lines in the top row connect control data with the next available stimulation data point (there were no correct saccades starting during −120 and −80 stimulation periods because this would abort fixation). ***C***, Summary of RT effects for contraversive (left) and ipsiversive (right) saccades combined for the two monkeys and all vertical target positions (mean and SE across sessions). Top and bottom rows show saccades that started either during the stimulation period or after stimulation offset. Note that the −40 ms period is a special case in which the separation into during and after stimulation does not provide meaningful information because the offset of this stimulation period (160 ms after the Go signal) happens at the same time as the mean onset of saccades in the control condition (165 ± 2 ms and 165 ± 2 ms; contraversive and ipsiversive saccades, respectively, with both monkeys combined). Therefore, saccades that started during the −40 ms stimulation period would by definition seem facilitated compared with control and saccades that started after stimulation would appear delayed. The RTs in all stimulation periods were compared with control trials (marked as “C”) using Kruskal–Wallis followed by Mann–Whitney *U* test, Bonferroni corrected (*p < 0.05, **p < 0.01).

Figure 4 quantifies RT effects across sessions. In control trials (gray), both monkeys had comparable RTs with unimodal distributions. The RT distributions for different stimulation periods confirmed that the stimulation predominately delayed the saccade initiation (Fig. 4*A*, top, in each monkey). The effect reached significance in a large proportion of individual sessions (Fig. 4*A*, bottom). Two distinct modes were evident upon inspection of RT distributions in Go and late period stimulation trials. As illustrated in examples shown in Figure 3*C*, the first mode contained saccades that started during the stimulation train, the second mode included saccades that started after the stimulation offset. Both effects were present in both monkeys, although Monkey L had fewer sessions in which the second mode was evident, especially for contraversive targets (+40 ms period: three sessions in Monkey L, 14 sessions in Monkey C; +80 ms period: one session in Monkey L, nine sessions in Monkey C). Interestingly, there was a correlation between the depth of the microstimulation site and the probability of ipsiversive deferred saccades in both monkeys, suggesting that the occurrence of deferred saccades is site specific (but not monkey specific). In the subsequent analysis, we separated the saccades into these two categories (“during stimulation” and “after stimulation”) and calculated a mean RT for each stimulation period across trials in each session and then across sessions (Fig. 4*B*,*C*). Figure 4*B* plots the data separately for each monkey and for each vertical target position, demonstrating the consistency of RT effects. Monkey L showed weaker delays for saccades that started during stimulation (Fig. 4*B*, top row), especially for contraversive instructed trials, but even in his data, the delay was significant across sessions (contraversive instructed: +40 ms simulation period, *p* < 0.05; ipsiversive instructed: Go, +40 ms, +80 ms stimulation periods, *p* < 0.01; Kruskal–Wallis followed by Bonferroni-corrected Mann–Whitney *U* test). Sessions with saccades that were deferred until after stimulation offset were also present in both monkeys (Fig. 4*B*, bottom row). Therefore, in Figure 4*C*, we combined data from both monkeys.

Across all vertical target positions, saccades that started during stimulation were delayed by 10–26 ms (minimum to maximum) in the Go and late stimulation periods, with a maximal delay occurring in the +40 ms or +80 ms stimulation periods. The saccades with onsets that were deferred until the end of the stimulation were initiated 35 ± 2 ms (contraversive) and 42 ± 2 ms (ipsiversive) after the stimulation offset (37 ± 2 ms and 48 ± 1 ms in Monkey C and 30 ± 6 ms and 33 ± 3 ms in Monkey L).

The main difference between the effects in the two visual hemifields was the RT facilitation, present only for ipsiversive saccades in early stimulation periods (−120 ms and to a lesser extent −80 ms), which all fell in the “after stimulation” category (Fig. 4*C*). This ipsiversive facilitation (16 ± 3 ms in the −120 ms period) was evident in the RT distributions (cf. gray and blue distributions), was significant in 14 of 15 sessions in Monkey C and in 8 of 15 sessions in Monkey L, and was significant in each monkey across sessions (*p* < 0.01 Monkey C, *p* < 0.05 Monkey L, Friedman test with *post hoc* Wilcoxon signed-rank test). Another difference between the effects in the two hemifields was that, whereas RT delays followed a similar pattern for contraversive and ipsiversive saccades, the effect was stronger for the ipsiversive side (individually in each monkey, *p* < 0.05 for all late stimulation periods, Friedman test with *post hoc* Wilcoxon signed-rank test, Bonferroni corrected). For example, in the +80 ms stimulation period, the delay was 46 ± 10 ms for contraversive and 83 ± 11 ms for ipsiversive saccades (*p* < 0.01). This observation will be considered when looking at the choice behavior (see below).

**Figure 5.**
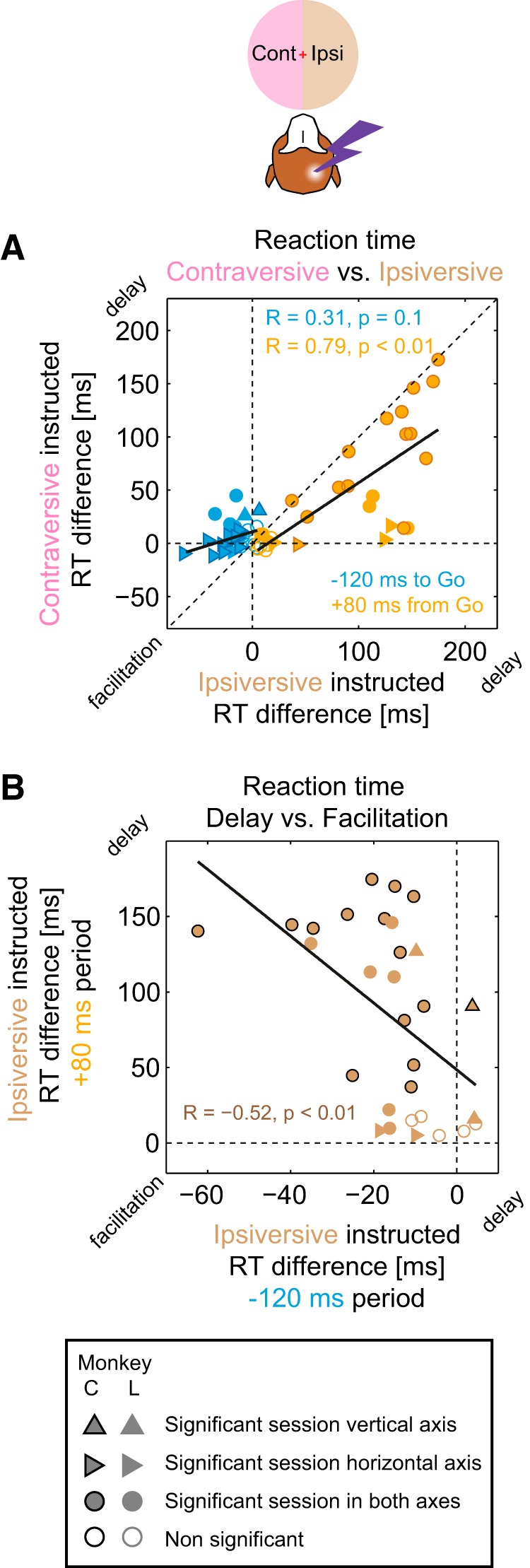
RT correlations across sessions. Two stimulations periods, −120 ms before and 80 ms after the Go signal, in which RT effects (facilitation and delay, respectively) were overall strongest and were selected for the correlation analysis. Data from both monkeys are combined in this and subsequent correlation plots. Filled circles indicate sessions in which both effects were significant, triangles indicate that only one of the effects was significant, and open circles indicate sessions with no significant change for any of them. ***A***, Contraversive versus ipsiversive RT difference (stimulation − control) in each session. For the early stimulation period −120 ms before the Go signal (blue symbols), stronger facilitation (negative RT difference) in ipsiversive trials had an insignificant trend to correlate with shorter RTs in contraversive trials. For the late stimulation period +80 ms after the Go signal (orange symbols), delays (positive RT difference) in ipsiversive and contraversive trials were correlated, with most data points below the main diagonal (ipsiversive delay > contraversive delay). ***B***, RT delay versus facilitation in ipsiversive saccades in each session. The facilitation due to stimulation in the −120 ms period and the delay due to stimulation in the +80 period ms were correlated. Black lines show best linear fits.

The relationship of RT delays between contraversive and ipsiversive saccades is further illustrated by the scatter plot of ipsiversive versus contraversive delays in the +80 ms stimulation period (RT difference, stimulation minus control) across sessions, showing a strong correlation between the two delays (Fig. 5*A*; Spearman's *r* = 0.79, *p* < 0.01). No significant contraversive–ipsiversive correlation was found for RT effects at −120 ms stimulation period.

We also investigated whether there was a relationship between facilitation and delay effects across sessions in two representative early (−120 ms) and late (+80 ms) stimulation periods for ipsiversive saccades that showed both effects. Indeed, there was a strong correlation between the facilitation and the delay (Fig. 5*B*; Spearman's *r* = −0.52, *p* < 0.01). Sessions that showed more facilitation in the early stimulation period also had more delay in the late stimulation period, indicating a shared influence of session-by-session variations in stimulation effectiveness. This relationship is in contrast to the opposite effect (less facilitation, more delay) found in the caudate nucleus (Watanabe and Munoz, 2011).

Finally, very similar effects on saccadic RTs were found for choice trials, including the facilitation of ipsiversive saccades in early stimulation periods (Fig. 4) and a larger delay for ipsiversive choices compared with contraversive choices (+80 ms stimulation period: 55 ± 10 ms contraversive, 83 ± 12 ms ipsiversive, *p* < 0.01, Kruskal–Wallis with *post hoc* Mann–Whitney *U* test, Bonferroni corrected).

### Visually-guided task: time-dependent spatial choice modulation

**Figure 6.**
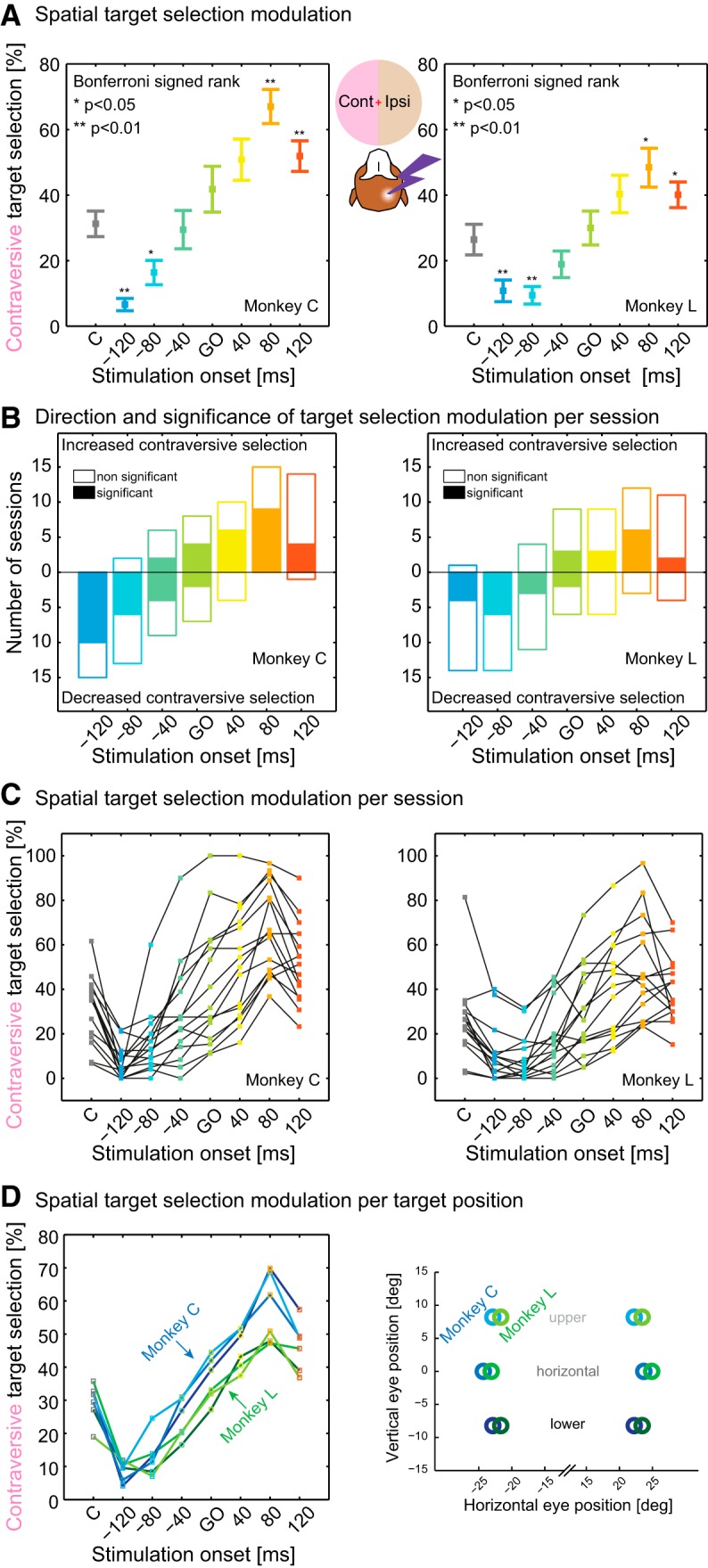
Effect of stimulation on target selection in visually-guided saccade task. ***A***, Percentage of contraversive target selection as a function of stimulation periods. In control trials (marked as “C”), both monkeys showed an ipsiversive (right) target selection bias despite initial bias equalization (see Materials and Methods). In stimulation trials, current applied before the Go signal further decreased the selection of contraversive targets. Late stimulation periods increased contraversive target selection. Mean and SE across sessions, *p-*values from Friedman test followed by Wilcoxon signed-rank test, Bonferroni corrected (*p < 0.05, **p < 0.01). ***B***, Target selection modulation per session: direction and significance. For each session, we used Bonferroni-corrected Fisher's exact test to compare target selection in stimulation trials to control trials for each stimulation period. The direction of the effect is shown with a positive or negative vertical bar corresponding to increased or decreased contraversive selection; statistically significant sessions are filled. ***C***, Percentage of contraversive target selection as a function of stimulation periods in individual sessions. Black lines connecting dots link data points from individual sessions. ***D***, Target selection modulation per vertical position of left/right target pairs (right). The inset on the left shows the corresponding color code (Monkey C, blue; Monkey L, green; light-, medium-, and dark-shaded traces denote upper, horizontal, and lower target positions).

In agreement with predictions from our previous pulvinar inactivation results (Wilke et al., 2010, 2013), dPul stimulation increased contraversive target selection, but only in late stimulation periods in which the train was delivered after the target onset/Go signal, during the decision and motor preparation phase (Fig. 6*A*). Surprisingly, stimulation in early periods, which started before but ended after the target onset, led to a decrease in contraversive selection (Fig. 6*A*). This biphasic modulation of spatial choice preference was a consistent pattern across sessions (Fig. 6*B*,*C*), showing maximal ipsiversive bias in the −120 ms or −80 ms periods and maximal contraversive bias in the +80 ms period. Furthermore, this pattern was consistent across upper, horizontal, and lower vertical target positions (Fig. 6*D*).

Given the resemblance of the choice effect to the modulation of ipsiversive RTs (first facilitation, then delay), we investigated whether time courses of changes in RTs and target selection across stimulation periods were similar. To this end, we correlated the mean percentage of contraversive selection with the mean ipsiversive choice RT, across stimulation periods (the ipsiversive choice RT was chosen to comprise both delay and facilitation RT effects), and found a strong linear correlation (Spearman's *r* = 0.99, *p* < 0.001, Monkey C; *r* = 0.89, *p* = 0.012, Monkey L; a similar effect was found for the correlation with ipsiversive instructed RTs: *r* = 0.96, *p* = 0.003, Monkey C; *r* = 0.93, *p* < 0.001, Monkey L). This demonstrates the temporal congruency of choice and RT effects: in early stimulation periods, the ipsiversive choice bias was accompanied by the (ipsiversive) RT facilitation and, as stimulation onsets progressed toward the later decision and motor planning phases of a trial, the contraversive choice bias was accompanied by the RT delay.

**Figure 7.**
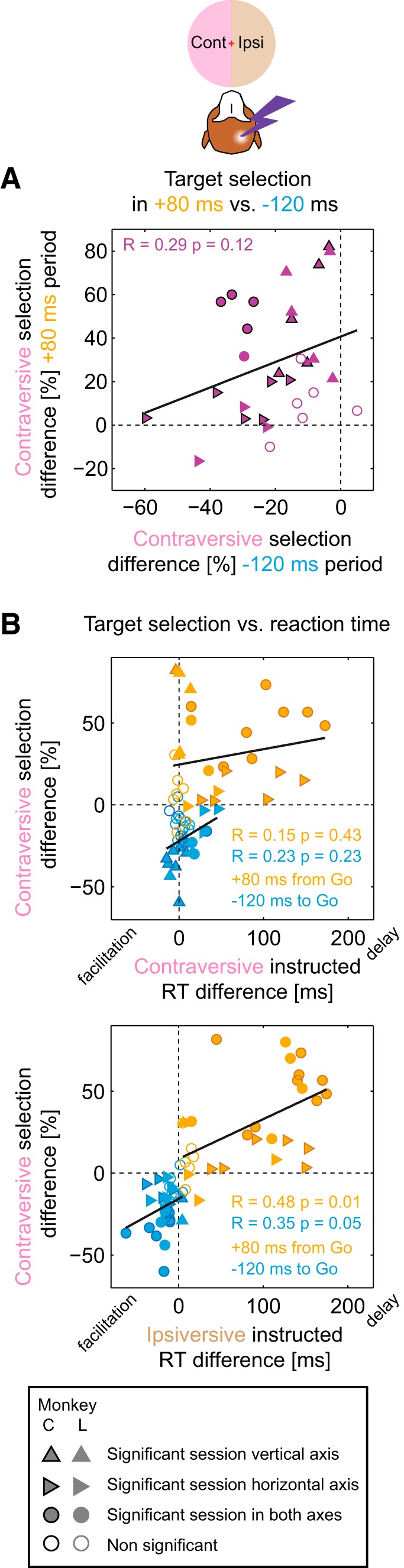
Target selection and RT correlations across sessions. ***A***, Contraversive target selection difference (stimulation − control) in −120 ms vs +80 ms stimulation periods in each session. There was a weak, insignificant correlation between contraversive bias at the +80 ms period and ipsiversive bias at the −120 ms period. ***B***, Contraversive target selection difference versus RT difference for the −120 ms and +80 ms stimulation periods for contraversive (top) and ipsiversive (bottom) saccades in each session. There was a weak, insignificant correlation between ipsiversive bias and RT changes in the −120 ms period (blue symbols) for both contraversive and ipsiversive saccades and a strong correlation between contraversive bias and RT delay in the +80 ms period only for ipsiversive saccades (orange symbols).

To investigate whether ipsiversive bias in early stimulation periods and contraversive bias in late stimulation periods might represent manifestations of the same neural mechanism, we correlated the strength of both effects across sessions. For the early −120 ms period, we found only an insignificant tendency for a stronger ipsiversive bias to be associated with a weaker contraversive bias in the late +80 ms period (Fig. 7*A*; Spearman's *r* = 0.29, *p* = 0.12). We also tested the −80 ms period instead of −120 ms period and found a stronger positive correlation (Spearman's *r* = 0.65, *p* < 0.01). Note that the positive correlation signifies an inverse relationship between strength of early ipsiversive and late contraversive bias. Therefore, at least on a session-by-session level, the relationship between the strength of the two effects is not straightforward, suggesting that factors other than overall stimulation effectiveness, for example, variations of session-specific spatial preferences, might play a role. Notably, this is the only aspect we found to be incongruent between directions of the across-sessions trends for RT and choice: recall that, for the ipsiversive RT, a stronger early facilitation was associated with a stronger, not weaker, late delay (cf. Fig. 5*B*).

It is important to emphasize, however, that most sessions exhibited a biphasic course of choice modulation (Fig. 6*C*) and, in five sessions, both effects reached significance even after the conservative Bonferroni correction for all seven stimulation periods (Fig. 7*A*). Therefore, the biphasic choice modulation illustrated in Figure 6*A* is not a consequence of averaging across sessions with either only early or only late period effects.

Similarly, to test the relationship between the choice bias and the RT effects, we correlated the stimulation-induced changes in the RT with the changes in choice preference across sessions. The increase of contraversive choices in the late +80 ms period correlated with the RT delay for ipsiversive instructed saccades, but not with the RT delay for contraversive instructed saccades (Fig. 7*B*; Spearman's *r* = 0.15, *p* = 0.43; *r* = 0.48, *p* = 0.01, for contraversive instructed and ipsiversive instructed RT, respectively). The same dependency was observed for choice RT delay (*r* = 0.09, *p* = 0.64; *r* = 0.50, *p* < 0.01, for contraversive choice and ipsiversive choice RT, respectively). Conversely, the decrease in contraversive choices in the early −120 ms period was only weakly and insignificantly correlated with the RT changes in both contraversive and ipsiversive instructed saccades. Therefore, the contraversive choice bias in the late periods was associated with stronger, likely subjectively undesirable, ipsiversive delays. This reasoning is further supported by strong positive correlations between the contraversive selection increase versus ipsiversive − contraversive RT difference in all but the two earliest stimulation periods (Spearman's *r* > 0.5, *p* < 0.01 for −40, Go, +40, and +120 periods, *r* = 0.44, *p* = 0.02 for +80 period).

We interpret the opposite direction effects in the early and the late stimulation periods as different manifestations of the same stimulation-induced mechanism. Decrease of contraversive choices and shortening of ipsiversive RTs suggest an ipsiversive orienting tendency due to the stimulation in the early periods starting before the Go signal. Conversely, increase of contraversive choices and stronger ipsiversive RT delay in the late stimulation periods point to a contraversive drive. To reconcile these findings, we propose that the effect of pulvinar activation is invariably contraversive and the apparent ipsiversive orienting is the consequence of a compensatory process that takes place due to behavioral task demands. In brief, when the stimulation is delivered in the early periods, while monkeys are tasked with maintaining fixation, they are (at least partially) suppressing or opposing the detrimental contraversive eye movements and this ipsiversive push-back against the stimulation-induced contraversive drive “spills over” beyond the stimulation offset to the interval after the Go signal when the decision and motor preparation take place. In agreement with this interpretation, but on a longer time scale across trials, in blocks of trials without stimulation, the contraversive target selection was higher than in the control (no stimulation) trials interleaved with the stimulation trials during stimulation blocks, suggesting that monkeys exhibited an ipsiversive tendency when “released” from stimulation (blocked-interleaved difference 14.6 ± 5.3%, *p* < 0.05 for Monkey C, 14.3 ± 6.5%, *p* < 0.01 for Monkey L, mean ± SE, Wilcoxon signed-rank test on differences). This and other alternative explanations are further considered in the Discussion.

### Visually-guided task: dPul versus vPul

**Figure 8.**
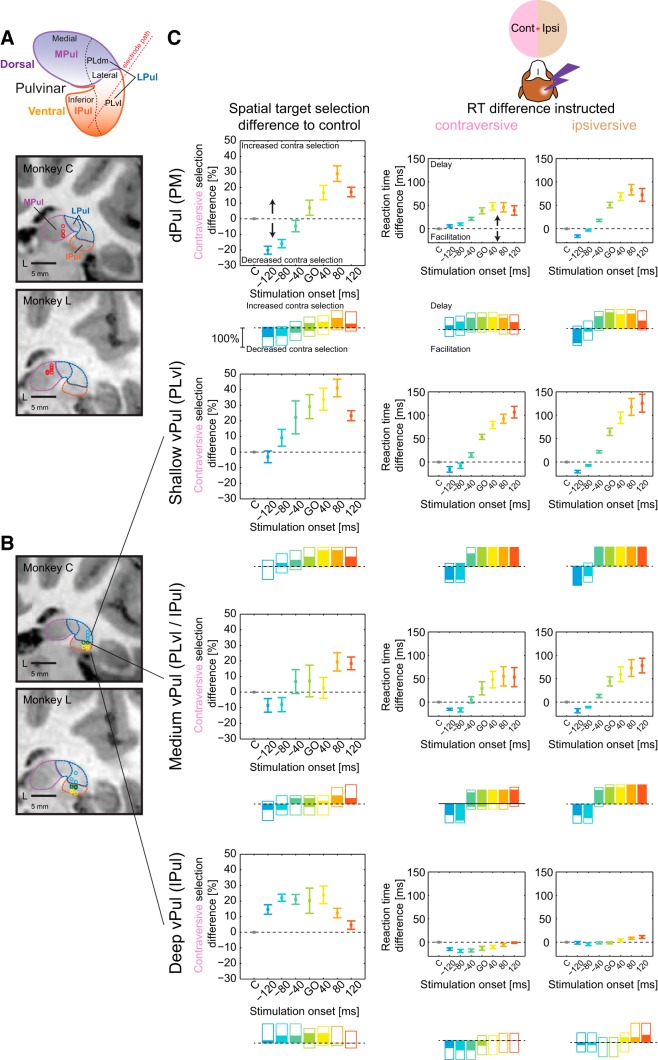
Summary of stimulation effects in dPul and vPul. ***A***, Top, Pulvinar parcellation schemes: dorsal versus ventral; medial, lateral, and inferior; with the lateral nucleus further subdivided to PLdm and PLvl. Bottom, Stimulation sites localization in the dPul (Monkey C: grid *y* = 5; Monkey L: grid *y* = 3), chamber normal coronal sections. ***B***, Stimulation sites localization in the vPul (Monkey C: grid *y* = 4; Monkey L: grid *y* = 3), chamber normal coronal sections. ***C***, In addition to 30 stimulation sites in dPul (top row), we performed control stimulations in 21 sites in vPul to assess the specificity of the stimulation effects on target selection and saccade generation. We binned these sites into three depth groups: shallow (six sessions, three in Monkey C and three in Monkey L), medium (seven sessions, two in Monkey C and five in Monkey L), and deep (eight sessions, five in Monkey C and three in Monkey L). Due to the angled chamber orientation, as we advanced deeper, we also targeted more medial and anterior parts of the vPul. One depth group is shown per row. For all rows, the left column, upper subpanel is the target selection difference from control, mean and SE across sessions and the bottom subpanel is the direction and significance of the stimulation effect per session normalized to the number of sessions (100% scale bar). The significance in each session was assessed using Fisher's exact test, Bonferroni corrected, filled colors (*p* < 0.05). Middle and right columns are data for instructed contraversive and ipsiversive RT effects, with significance in each session assessed using Kruskal–Wallis test followed by Mann–Whitney *U* test, Bonferroni corrected.

The effects of dPul stimulation were robust and consistent across multiple sites in both monkeys. To test for the site specificity of those effects, we conducted a series of control stimulation experiments in the vPul targeting different depths along the electrode track (Fig. 8*A*,*B*). Figure 8*C* summarizes the results of the experiments as a function of electrode depth. The stimulation in shallow vPul sites, which correspond to the PLvl according to the parcellation of Kaas and Lyon (2007), resembled the patterns obtained in the main dPul experiment, with the exception of bilateral (not only ipsiversive) RT facilitation and no clear ipsiversive choice bias in the early stimulation periods. Deeper sites (“medium vPul”) at the estimated border between the ventrolateral and inferior pulvinar showed similar but weaker stimulation effects. In contrast, deepest sites (“deep vPul”) in the inferior pulvinar exhibited a distinct pattern: a contraversive bias in all but very late stimulation periods, a contraversive RT facilitation (no delay) in the same periods, and only very small RT effects for the ipsiversive saccades. It is worth noting that, in 3 cases for Monkey C and 4 cases for Monkey L at least 2 different electrode depths were used in the same penetration and, on such days, the neighboring sites were separated only by 1–2 mm but still elicited distinct behavioral patterns. This suggests that the effects of stimulation were markedly localized to specific portions of surrounding tissue.

### Memory-guided task: dissociating cue processing and motor planning phases

**Figure 9.**
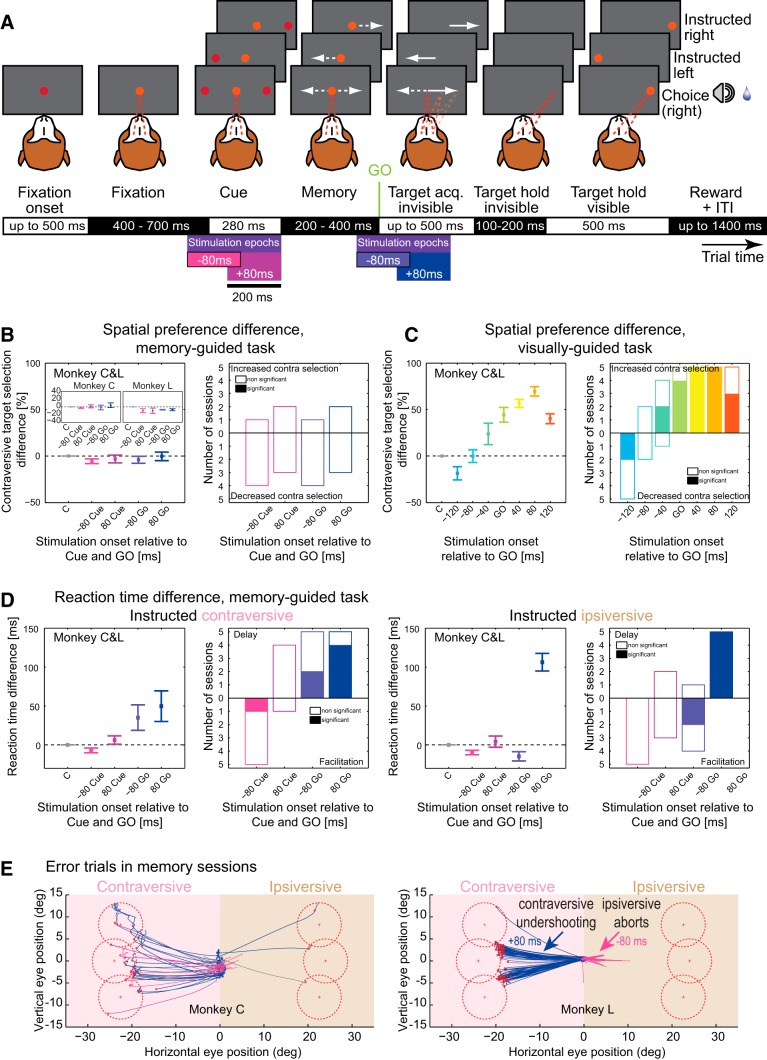
Stimulation effects in memory-guided saccade task (dPul). ***A***, Task layout. Stimulation was delivered in one of four periods: starting before onset of the visual cue(s) (−80 ms Cue), after the onset of the visual cue(s) (+80 ms Cue), before the Go signal (−80 ms Go), or after the Go signal (+80 ms Go). Trials without stimulation were interleaved as a control. ***B***, ***C***, Target selection in the memory-guided task (***B***) and in the visually-guided saccade task in the same sites and sessions (***C***) (five sessions and three sessions in Monkey C and two sessions in Monkey L), mean and SE of contraversive selection difference from control, and direction and significance of preference change from control (Fisher's exact test, Bonferroni corrected). Insets in ***B*** for the memory-guided task show data for each monkey. ***D***, Effect of stimulation on RT in the memory-guided saccade task for instructed contraversive (left) and ipsiversive (right) trials. For both hemifields, panels on the left show the mean and SE across sessions and panels on the right show direction and significance of effect per session; significance in each session was assessed by Kruskal–Wallis test with *post hoc* Mann–Whitney *U* test, Bonferroni corrected. ***E***, Eye position traces during instructed memory-guided saccade error trials, with two sessions combined for each monkey (Monkey C on the left and Monkey L on the right). Trajectories are colored according to the period in which stimulation occurred; trials in which no stimulation was delivered are gray. There were two periods in which both monkeys showed a considerable decrease in the hit rate (<80%; Table 3): before the Cue onset and after the Go signal. Errors in trials in which stimulation was delivered before the Cue onset were mostly fixation aborts toward the ipsiversive cue after the stimulation period ended. Errors after the Go signal were mostly hypometric saccades that did not reach the target window.

The late stimulation periods in the visually-guided task started during the visual target presentation concurrently with the ensuing decision and saccade planning. To assess whether the stimulation effect on choices was due to affected visual, decision, or motor processing stages, we used a memory-guided saccade task and delivered the stimulation to the dPul in four different trial periods: before cue onset, after cue onset, before the Go signal, and after the Go signal (Fig. 9*A*). As shown in Figure 9*B*, there was no effect on target selection in any of the stimulation periods, although stimulation in the same sites and sessions during the visually-guided task elicited a consistent biphasic choice bias as described above (Fig. 9*C*). At the same time, and consistent with the visually-guided task, memory saccade RTs in the contraversive space were delayed with stimulation before and especially after the Go and the ipsiversive saccades were facilitated by the stimulation before the Go and strongly delayed by the stimulation after the Go (Fig. 9*D*).

In addition, the stimulation affected the memory-guided task hit rates (Tables 3, 4). Specifically, the two conditions in which the hit rate dropped <80% in the stimulation trials in both monkeys were as follows: (1) instructed ipsiversive trials when the stimulation started before cue (−80 ms cue) and (2) instructed contraversive trials when the stimulation was applied after the Go signal (+80 ms from Go). Error trial eye position trajectories (Fig. 9*E*) showed that most errors in the instructed ipsiversive trials were fixation aborts due to saccades toward the ipsiversive cue (79% of error trials with the stimulation onset before the cue), with a latency of 74 ± 14 ms (mean ± SD) after stimulation offset. The same effect was observed in choice trials; even in the presence of two opposite cues, monkeys tended to break fixation by making saccades to the ipsiversive side (88% of error trials with the stimulation onset before the cue). Therefore, monkeys had difficulty suppressing reflexive saccades to the ipsiversive cues after stimulation offset, which is consistent with our interpretation of ipsiversive orienting in the early stimulation periods in the visually-guided task. Note that this does not contradict the absence of stimulation effect on choices: if the fixation was maintained and thus trials were not aborted, then subsequent choices were not affected.

**Table 3.** Hit rates in the memory-guided saccade task, instructed trials, dorsal pulvinar stimulation (mean ± SE across sessions)

Stimulation period onset	Contraversive hit rate (%)			Ipsiversive hit rate (%)		
	Both monkeys	Monkey C	Monkey L	Both monkeys	Monkey C	Monkey L
Control (no stimulation)	96 ± 2	93 ± 3	100 ± 0	89 ± 8.3	82 ± 13	100 ± 0
−80 ms to Cue onset	94 ± 5	90 ± 7	100 ± 0	73 ± 9*	*74* ± *14*	*71* ± 11*
+80 ms from Cue onset	96 ± 2	94 ± 3	98 ± 2	91 ± 8	84 ± 13	100 ± 0
−80 ms to Go	89 ± 5	87 ± 7	92 ± 8	84 ± 15	*74* ± *25*	98 ± 2
+80 ms from Go	53 ± 14*	52 ± 22*	54 ± 21*	89 ± 7	84 ± 12	97 ± 0

In two stimulation periods, −80 ms to ipsiversive cue onset and +80 ms from Go in contraversive trials, there was a drop in performance <80% (in bold and with asterisk, significant in at least one session, Fisher's exact test, Bonferroni corrected, *p* < 0.05). Hit rate drops <80% that did not reach significance are in italics.

**Table 4.** Hit rates (a fraction of successfully completed trials regardless of chosen hemifield) in the memory-guided saccade task, choice trials, dorsal pulvinar stimulation (mean ± SE across sessions)

Stimulation period onset	Hit rate (%)		
	Both monkeys	Monkey C	Monkey L
Control (no stimulation)	92 ± 4	89 ± 6	97 ± 2
−80 ms to Cue onset	76 ± 4*	78 ± 7*	72 ± 1*
+80 ms from Cue onset	93 ± 6	88 ± 10	99 ± 1
−80 ms to Go	89 ± 6	85 ± 9	95 ± 0
+80 ms from Go	74 ± 15*	67 ± 25*	86 ± 4*

Similar to instructed trials, in two stimulation periods, −80 ms to cue onset and +80 ms from Go, there was a drop in performance (in bold and with asterisk, significant in at least one session, Fisher's exact test, Bonferroni corrected, *p* < 0.05). Note that because these were two-target free-choice trials, we did not assign aborted, incomplete trials to left or right choices; therefore, here, the trials are not divided into contraversive and ipsiversive. However, the plot of eye position trajectories in error trials, similar to Figure 9*E* for the instructed trials, demonstrated similar effects: saccades to the ipsiversive cue after the offset of early −80 ms stimulation and contraversive undershooting in +80 ms from Go late stimulation period (plot not shown).

Most errors in the second condition, the contraversive trials, were due to contraversive undershooting (85% of instructed and 64% of choice error trials with the stimulation onset after the Go signal). Monkeys were more severely affected by the stimulation during the motor preparation and response phase when there were no visible targets to guide it compared with the visually-guided task, which showed milder hypometria with no drop in hit rates (cf. Fig. 3*B*, Table 2). But even taking into account those undershooting error choice trials that were directed toward contraversive targets, the choice was not significantly modulated by the stimulation in +80 from Go period in any of the sessions (*p* > 0.05, Fisher's exact test).

Given the lack of choice effects, we considered the possibility that the delayed RTs are a consequence of stimulation interfering with the processing of fixation point offset (i.e., Go signal), but we deem it unlikely given the similarity to RT delays in the visually-guided task (where peripheral target onset coinciding with the fixation offset served as even more apparent Go signal) and the spatially specific difference between contralateral and ipsilateral RT delays (ipsiversive delay > contraversive delay).

These results give rise to several important implications. First, the choice-relevant aspects of cue processing seem unaffected when they are temporally dissociated from the motor response. Similarly, the stimulation does not seem to affect the choice when the decision can be formed in advance of the action, neither in the cue/memory period nor just before or during the motor response. Third, the RT and the choice effects, which were largely congruent in the visually-guided task, were dissociated in the memory-guided task and thus might not depend critically on each other. This dissociation is reminiscent of recent perceptual decision study in the caudate (Ding and Gold, 2012). Like basal ganglia, the pulvinar is involved in multiple functional loops (Sherman and Guillery, 2002) and different populations or pathways might encode distinct processes. Together, the results of visually-guided and memory-guided tasks indicate that the transient pulvinar stimulation contributes to the spatial decision process only when the choice must be formed and executed close in time.

### Neuronal properties in the dPul

To better understand the neural contribution of dPul to the behavioral effects of microstimulation, we analyzed the activity of 230 dPul units recorded in the visually-guided saccade task and 365 dPul units in the memory-guided saccade task in and around the same stimulation sites (see Materials and Methods). Dorsal pulvinar units predominantly showed low firing rates (mean firing rate across all task periods: 10 and 11 spikes/s, median: 6 and 7 spikes/s, SD: 10 and 11 spikes/s, for the visually-guided and memory-guided task, respectively).

**Figure 10.**
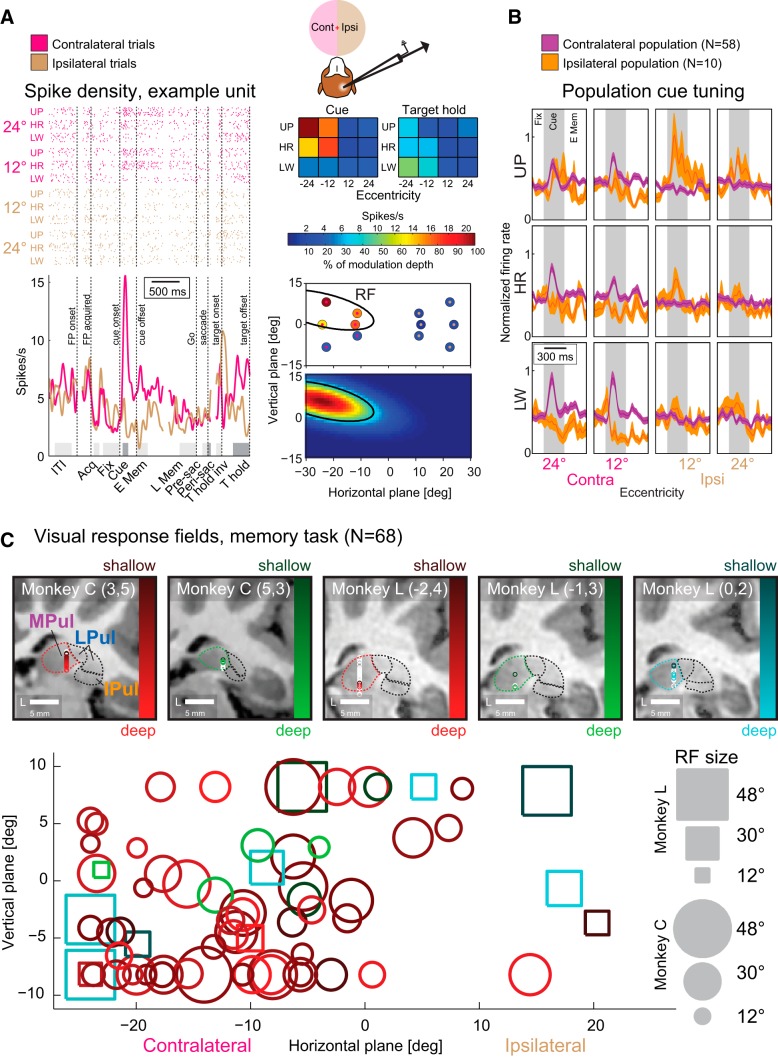
Spatial RF properties in the dPul. ***A***, Offline RF estimation in an example unit (Cur_20150617_03). Left, Raster plot and resulting spike density functions (SDFs) shown separately for contralateral and ipsilateral trials in magenta and brown, respectively. Trials are grouped by hemifield, eccentricity, and vertical target position, upper (UP), horizontal (HR), and lower (LW). For the raster plot and SDF, black dotted lines denote events: fixation point onset (“FP onset”), acquiring fixation (“FP acquired”), cue onset, cue offset, and beginning of the memory period, offset of the central fixation point (which also served as the Go signal, “Go”), saccade onset, and onset and offset of the peripheral target. Discontinuous traces indicate gaps in alignment to events: FP onset, cue onset, saccade onset, and target offset; the other event markers denote average onset relative to alignment events. Gray boxes above the time axis indicate analyzed epochs (see Materials and Methods). Top right, Average firing rates for all 12 target positions during the cue and target hold epochs (top part of the color scale). Bottom right, Modulation depth and Gaussian fit defining the RF for that unit. Percentage modulation depth of cue responses is displayed for each target location at its actual position on the screen (bottom part of the color scale). The size of the visual stimuli is indicated by the dot in the center of each target (0.5° radius). The superimposed ellipse represents the boundaries of the Gaussian fit (2 SDs to each side; see Materials and Methods). For this unit, the RF size was estimated as 21°. ***B***, Mean population response and SE across units during fixation hold, cue, and early memory epochs for ipsilateral and contralateral subsets of units, shown in orange and purple, respectively, for each target position. The two subsets represent units that had a main effect of target position during the cue epoch (gray shaded area) and were sorted into contralateral and ipsilateral populations according to the preferred hemifield. Before averaging across units, the mean peak of each unit's activity during the cue epoch across all trials to the preferred hemifield was normalized to 1. ***C***, Visual RFs in the memory-guided saccade task. Top, Electrode tip position in individual recording sites (circles) in chamber-normal coronal sections corresponding to specific grid location (*x*, *y*; in parentheses). Recording sites where no spatially tuned units were found are denoted by white circles; recording sites that showed tuning are shown in red, green, and blue colors representing different grid locations, with dark-to-light shades denoting recording depth. Pulvinar nuclei outlines as in Figure 1, colored outlines are the MPul. Bottom, RF centers and sizes for all units showing a main effect of cue location. RF centers correspond to the center of markers: circles for Monkey C, and squares for Monkey L. The marker size represents RF size, scaled 10:1. The color of the markers indicates the recording sites corresponding to the site reconstruction panels above.

Visual RFs were estimated offline using an array of 12 target positions (12° and 24° eccentricity). Cue responses for each target position in the memory-guided saccade task were fitted with a 2D Gaussian profile (see Materials and Methods). The position of the Gaussian peak and the area covered by two Gaussian SDs to each side defined center and size of the RF. Figure 10*A* illustrates firing patterns and RF estimation in one example unit. Here, we refer to the (visual) RFs as those computed in the cue epoch, but RFs could also be computed during eye movements and peripheral fixation. As can be seen in the example for the peripheral target hold epoch in Figure 10*A*, postsaccadic RFs can differ from visual RFs, as has been reported in the PLdm (Robinson et al., 1986).

The RF estimation was performed on all units showing a main ANOVA effect of stimulus position during the cue period (68 units). We separated this subset further by each unit's preferred hemifield and looked at the population cue response for each of the 12 target positions in contralaterally and ipsilaterally tuned subsets (Fig. 10*B*). Even though the contralateral subset was much larger (58 units) than the ipsilateral subset (10 units), contralateral population cue response was more time locked than ipsilaterally tuned responses. In addition, contralateral responses seemed to be more consistent across targets, with a small preference for lower and more peripheral targets.

Figure 10*C* illustrates the estimated RFs at a scale of 1:10 in a plot representing the visual target array field, with colors representing different recording sites. RF centers were scattered across the entire tested visual field and their size varied substantially. Note that, because RF centers were constrained to the dimensions of the target array, it may seem as if many RFs are clustered along those borders. However, our mapping and fitting approach did not allow drawing conclusions about potential RF centers outside of the target array. Typically, RF estimates were large (28 ± 9°) and most of them had their centers in the contralateral hemifield (mean eccentricity = 10° in the contralateral hemifield, median = 11°, SD = 11°), with a tendency for lower peripheral positions. We did not find a consistent topographical organization along the electrode recording tracts, like the previous dPul study (Petersen et al., 1985). The lack of retinotopic organization is consistent with fairly uniform microstimulation effects across sites and target positions. Furthermore, largely horizontal directions of small evoked saccades in Monkey C might have resulted from a vector summation of upward, horizontal, and downward RFs with a contralateral bias, possibly similar to a population coding in the deeper layers of the SC (Lee et al., 1988).

**Figure 11.**
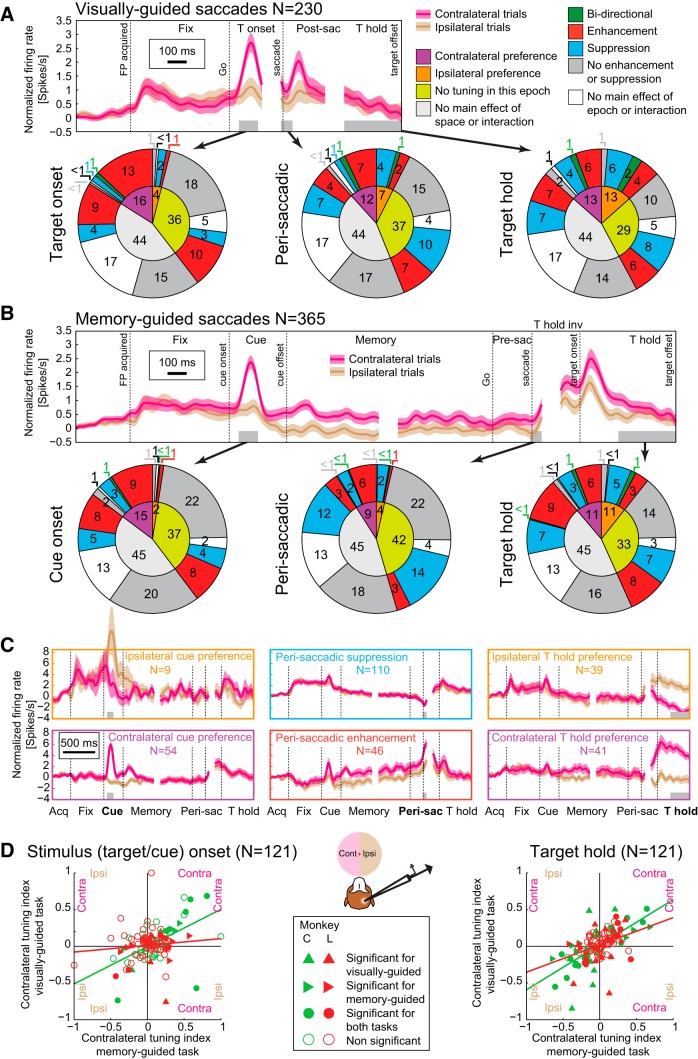
Neuronal population properties in the dPul. ***A***, Units recorded during the visually-guided saccade task (*n* = 230). Top, Average baseline-corrected firing rate, mean (solid traces) and SE (shaded bands) across units, separately for contralateral and ipsilateral trials shown in magenta and brown, respectively. Same convention for alignment lines as in Figure 10*A*. Bottom, Spatial tuning and firing rate modulation in the three epochs (target onset, perisaccadic, and target hold). In each plot, sectors of the inner circle display the percentage of units that, in the respective epoch, preferred the ipsilateral hemifield (orange), contralateral hemifield (purple), were not tuned (light green), or were not tested for spatial tuning because they showed neither a main ANOVA effect of hemifield nor an interaction of hemifield and epoch (light gray). The outer sectors display the percentage of units that showed enhancement or suppression in the respective epoch compared with the fixation hold epoch for each of the four aforementioned subsets separately: units showing enhancement for one hemifield and suppression for the other (green), only enhancement (red), only suppression (blue), neither enhancement nor suppression (dark gray), or neither a main ANOVA effect of epoch nor interaction of epoch and hemifield and thus not tested for enhancement or suppression (white). ***B***, Similar to ***A***, but for the memory-guided saccade task. Top, Average baseline-corrected firing rate. Bottom, Spatial tuning and firing rate modulation in the three epochs (cue onset, perisaccadic, and target hold). ***C***, Average baseline-corrected PSTHs across different subsets of units in the memory-guided task. The subsets were defined by the classification in ***B***: ipsilateral cue tuning (top left), contralateral cue tuning (bottom left), perisaccadic suppression (top center), perisaccadic enhancement (bottom center), ipsilateral tuning during target hold (top right), and contralateral tuning during target hold (bottom right). ***D***, CIs (see Materials and Methods) for visually-guided versus memory-guided saccades for each unit where data for both tasks was available (*n* = 121). Filled markers denote units with significant tuning (see legend). Units recorded in Monkey C and L are in green and red, respectively. Left, CIs in “stimulus onset” (“target onset”/“cue onset”) epochs. Right, CIs in “target hold” epoch. Lines indicate best linear fits.

In the visually-guided task, there was also a stronger population response for contralateral stimulus onset (target onset epoch, “T onset”; Fig. 11*A*). In addition, population response showed a transient and then sustained enhancement after central fixation acquisition (“Fix”) and transient postsaccadic peak (“Postsac”), which was stronger for contralateral than for ipsilateral targets. Note that the weak perisaccadic population response is due to different subsets showing either perisaccadic enhancement or suppression (see below).

Overall, 78% of units were modulated by the visually-guided task (main effect of epoch or epoch × hemifield interaction) and 56% showed spatial specificity in at least one epoch (main effect of hemifield or epoch × hemifield interaction). Epoch-specific enhancement or suppression, relative to the fixation baseline, was analyzed for the first subset (78%) and epoch-specific spatial tuning was analyzed in the latter subset (56%). The bottom panels in Figure 11*A* summarize the main patterns of spatial tuning and enhancement/suppression in the three epochs: “target onset,” “perisaccadic,” and “target hold.” The significant tuning was predominantly to the contralateral hemifield in the “target onset” epoch, but became more equalized in “perisaccadic” and especially in “target hold” epochs. Spatially tuned units showed predominantly enhancement of firing relative to the fixation baseline (red outer sectors) for target onset epoch, whereas all other subsets had more equal proportions of enhancement and suppression.

Population response in the memory-guided saccade task (Fig. 11*B*) additionally revealed preference for contralateral trials during the memory period and in the postsaccadic peripheral fixation epoch in absence of visual stimulus, before target onset (“target hold invisible”). Similarly to in the visually-guided task, 84% of units were task modulated (main effect of epoch or epoch × hemifield interaction) and 55% showed a main effect of hemifield or epoch × hemifield interaction. Epoch-specific enhancement or suppression relative to the fixation baseline was analyzed for the first subset (84%) and the epoch-specific spatial tuning was analyzed in the latter subset (55%). The bottom panels in Figure 11*B* summarize the main patterns of spatial tuning and enhancement/suppression in the three epochs: “cue onset,” “perisaccadic,” and “target hold.” Again, the significant tuning was predominantly to the contralateral hemifield in the “cue onset” epoch, but it became more equalized in “perisaccadic” and especially in “target hold” epochs. Units that were contralaterally tuned in the cue epoch predominantly showed enhancement of firing relative to the fixation baseline. In addition to spatially tuned responses, an additional 47 units (13%) showed robust cue-related enhancement that was not spatially selective (no main effect of target position and no hemifield tuning). Nonspatially tuned units in the perisaccadic epoch showed predominantly suppression, whereas all other subsets had more equal proportions of enhancement and suppression (Fig. 11*B*).

To further assess the differences in spatial tuning in “cue onset” and “target hold” epochs, population responses for subsets that showed significant tuning in those epochs were derived (Fig. 11*C*, left and right columns). The units that were contralaterally tuned in the cue epoch on average did not show spatial tuning in the postsaccadic and the target hold epochs, suggesting that the tuning in the latter intervals can be congruent or incongruent with the visual cue tuning. This is further evidenced by weak contralateral cue tuning in both subsets that showed significantly tuned, either ipsilateral or contralateral, target hold response (Fig. 11*C*, right column).

A closer look at full-trial population responses for units showing perisaccadic suppression (110 units, 30%; Fig. 11*C*, middle column, top row) revealed that many of these units increased firing during central fixation (58 of 110). Those responses might resemble so called “fixation cells” reported in the FEF and in the SC (Munoz and Wurtz, 1993a; Izawa et al., 2009). However, recent work by Hafed and Krauzlis (2012) demonstrated that, at the level of the SC, the tonic activity during fixation encodes fixational microsaccades or a retinal error by neurons tuned to foveal locations of very small eccentricity; it remains to be seen whether some pulvinar neurons might be similarly related to fixation maintenance. Furthermore, many “fixation response” cells in our sample showed a decreased firing during the memory delay compared with the initial fixation (45 of 100 units that showed enhanced firing during fixation relative to ITI), suggesting that spatially specific aspects such as visual memory or motor planning can modulate their activity (Fig. 11*C*, middle column, top row). Conversely, units that showed perisaccadic enhancement (Fig. 11*C*, middle column, bottom row) also exhibited an increased firing in contralateral trials from the cue onset and during the memory delay, ramping up before and peaking soon after the saccade, similar to visuomotor neurons in frontoparietal areas.

To summarize spatial tuning properties, we calculated contralateral tuning indexes (CIs) for stimulus onset and peripheral fixation (“target hold”) responses for both tasks. Figure 11*D* shows distribution of CIs for the subset of units that were recorded in both tasks for each monkey. For both tasks, across all recorded units CIs were significantly positive (i.e., contralateral) during “stimulus onset” epochs (visually-guided task: 0.07 ± 0.24; memory-guided task: 0.09 ± 0.26; *p* < 0.001, two-tailed one sample *t* test); for the subset recorded in both tasks, only memory-guided task indexes were significantly positive (visually-guided task: 0.03 ± 0.23, *p* = 0.13; memory-guided task: 0.05 ± 0.26, *p* < 0.05). There was no significant tuning across the sample in the target hold epoch, reflecting nearly equal contralaterally and ipsilaterally tuned populations. For both epochs, there was a correlation between tuning indexes in the two tasks (Spearman's *r* = 0.35, *p* < 0.0001 for “stimulus onset” and *r* = 0.59, *p* < 0.0001 for “target hold), indicating that the spatial response properties in these two epochs are largely consistent across the two tasks.

Although the full analysis of complex neuronal properties in the recorded population is beyond the scope of the present study, these data provide several points aiding the interpretation of the stimulation results. First, most recorded neurons were modulated by the visual and/or oculomotor contingencies of the two tasks. Second, the overall contralateral tuning in response to the visual stimulus (e.g., target and cue onset epochs), as well as in the presaccadic (data not shown) and the perisaccadic epochs, is consistent with the contraversive drive elicited by the stimulation and the lack of topographic organization of RFs is consistent with similar stimulation effects across different sites and target positions. Third, the fact that the population tuning is still more contralateral than ipsilateral in the perisaccadic epochs suggests that the stronger RT delays for ipsiversive saccades in the late stimulation periods are not a direct consequence of disrupting ipsilaterally tuned populations more than contralaterally tuned ones. Fourth, a subset of units (cf. Fig. 11*A*,*B*, perisaccadic epochs, outer blue sectors corresponding to spatially nontuned populations and Fig. 11*C*, middle top panel) discharged vigorously during fixation intervals but paused firing in the perisaccadic period, potentially contributing to stimulation-induced saccade delays (Yang et al., 2008). Finally, many units had spatial tuning (both contralateral and ipsilateral) in the later part of the target hold period (starting at least 200 ms after the saccade offset, when the immediate postsaccadic effects are probably gone), suggesting a contribution of the dPul to the encoding of gaze, similar to the retinotopic inferior/lateral pulvinar (Robinson et al., 1990)

## Discussion

Electrical microstimulation of the dPul influenced selection and execution of goal-directed saccades in a spatially and time-dependent manner. This section focuses on the three main findings: (1) in the visually-guided task, stimulation starting before target onset (Go signal) reduced ipsiversive RTs, whereas stimulation at and after target onset caused a systematic increase in RTs for both ipsiversive and contraversive directions; (2) stimulation before the onset of targets increased ipsiversive choices and stimulation after onset of targets increased contraversive choices; and (3) in the memory-guided task, stimulation exerted effects on RT, but not on choices.

### Effects of microstimulation on saccade generation

Bilateral RT delays with microstimulation after the Go signal have been reported for structures involved in saccade control, such as the dlPFC (Wegener et al., 2008), FEF (Izawa et al., 2004a), supplementary eye field (SEF) and pre-SMA (Isoda, 2005; Yang et al., 2008), caudate (Watanabe and Munoz, 2010, 2011), and rostral SC (Munoz and Wurtz, 1993b). In cortex, delays were typically stronger for ipsiversive saccades (Izawa et al., 2004b; Isoda, 2005; Wegener et al., 2008), whereas the opposite pattern was observed in SC and caudate (Munoz and Wurtz, 1993b; Watanabe and Munoz, 2013). The delay can be interpreted as suppression of gaze shifting, facilitation of gaze holding, and/or inhibition of a mechanism that switches between the two behavioral modes and might be explained by direct or indirect, uncrossed and crossed projections to substantia nigra pars reticulata, SC, and/or brainstem saccade generator nuclei (Izawa et al., 2004b; Isoda, 2005).

Unlike SC and caudate and similar to frontal cortical areas, ipsiversive RT delays were stronger in the dPul, although ipsiversive and contraversive delays were correlated, suggesting a common mechanism; for example, the engagement of fixation neurons or nearly balanced recruitment of ipsilateral and contralateral populations, as well as untuned neurons.

In contrast to stimulation after the Go signal, stimulation starting before the Go shortened the ipsiversive RT, similarly to SEF and pre-SMA (Isoda, 2005; Yang et al., 2008) and caudate (Watanabe and Munoz, 2011). However, the above studies reported both contraversive and ipsiversive facilitation, indicating a general motor potentiation or release from gaze-holding signals. In caudate, release of SC/FEF from inhibition and subsequent rebound, or interplay between direct and indirect pathways, were suggested to explain the facilitation (Watanabe and Munoz, 2011). To account for the ipsiversive-specific RT facilitation in the dPul, we propose a different, directional mechanism, which is the same as that for the ipsiversive choice bias (see below).

### Effects of microstimulation on choices

Unlike the effects of microstimulation on saccade execution, interference on choices has been less studied, with most work focusing on perceptual decisions (Murasugi et al., 1993; Carello and Krauzlis, 2004; Hanks et al., 2006; Fetsch et al., 2014; Cicmil et al., 2015), but see (Opris et al., 2005; Mirpour et al., 2010). Therefore, one major question was whether and when pulvinar microstimulation influences free-choice target selection. Our previous work with pharmacological inactivation of dPul already implicated it in the spatial decision making (Wilke et al., 2010, 2013), but it was important to test whether the stimulation potentiates the “functioning” of the pulvinar, thus biasing choices in the direction opposite to the inactivation (i.e., contraversive vs inactivation-induced ipsilesional bias). Indeed, stimulation after the Go signal increased contraversive selection and, together with the contralateral neuronal tuning in the corresponding epoch, this suggests that it did not merely disrupt the normal functioning (Carello and Krauzlis, 2004). This is consistent with cortical and SC studies, which typically show a correspondence between the neuronal tuning and the direction of microstimulation effects (Clark et al., 2011).

However, even assuming a facilitatory activation by stimulation, the stimulation effects were not just a “mirror image” of inactivation. When stimulation started during fixation, before target onset, it caused ensuing ipsiversive bias, concurring with the ipsiversive RT facilitation. Such ipsiversive effects on target selection have been rarely reported. One study showed that microstimulation in upper layers of V1 biases choices away from the stimulated RFs (Tehovnik et al., 2002); similarly, caudate stimulation increased ipsiversive perceptual choices, away from contralateral RFs (Ding and Gold, 2012).

One hypothesis is that the ipsiversive selection is the manifestation of a stimulation-induced contraversive drive, which has to be counteracted during the fixation. Such a putative ipsiversive compensatory mechanism might be engaged until the end of the stimulation period and extend beyond the stimulation offset (after the Go signal) into the motor planning/execution epoch. Please note that this hypothesis does not necessarily imply that the monkeys were aware of the stimulation (Murphey and Maunsell, 2008) and compensated intentionally.

A related hypothesis is that the timing of stimulation offset relative to saccade RTs is important. Early stimulation periods ending ∼80 ms before the typical control RTs (∼160 ms) led to an ipsiversive “advantage” in RTs and choices, whereas stimulation periods overlapping with the RTs led to a contraversive bias. Although the stimulation causes a contraversive drive, the offset of the stimulation per se might trigger a transient ipsiversive rebound regardless of task requirements. To resolve whether the timing of stimulation onset or the stimulation duration/offset is the crucial factor for the ipsiversive facilitation, the duration of the stimulation trains should be varied systematically in future studies.

An even more mechanistic explanation might be that the time course of stimulation on the evoked activity is initially excitatory and then inhibitory (Histed et al., 2013). Therefore, the initial contraversive drive would be suppressed by the end of the early stimulation periods, during target selection. Indeed, inhibitory consequences of the thalamic stimulation on cortical activity have been reported (Logothetis et al., 2010) and a pulvinar stimulation study in anesthetized tree shrews found that evoked activity in extrastriate cortex consists of early and late waves, with a gap ∼200 ms after the stimulation onset (Vanni et al., 2015).

Another explanation might be that the dPul stimulation engages a contraversive attentional shift, which acts as a “cue” in the inhibition of return phenomenon (Dorris et al., 2002). There is also a possibility that the pulvinar fulfills distinct functions in different behavioral states, for example, filtering out contralateral distractors and inhibiting reflexive contraversive saccades (Van der Stigchel et al., 2010), until cortical inputs signal the initiation of the active motor preparation phase. In this case, the potentiation of pulvinar activity during fixation would lead to a suppression of the currently irrelevant contraversive space. The presence of fixation-like neurons discharging persistently when monkeys maintained fixation supports this notion. However, the occurrence of contraversive evoked saccades during fixation challenges this interpretation unless the motor effects can be completely dissociated from the attentional/target selection signals.

Although a combination of contraversive facilitation drive and ipsiversive compensatory/rebound effect after the early stimulation offset seems most parsimonious explanation for the observed effects, the question whether a given stimulation protocol leads to functionally beneficial enhancement of “normal” neuronal activation, to a functionally detrimental disruption, or to replacement or “hijacking” (Cheney et al., 2013) is a long-standing debate, relevant for all stimulation studies (Desmurget et al., 2013). Some of our stimulation effects are consistent with the disruption of the (contraversive) pulvinar processing: ipsilateral facilitation in early stimulation periods and delayed saccades. However, this hypothesis is hard to reconcile with the contraversive choice facilitation in later stimulation periods unless the apparent contraversive facilitation is the consequence of “less contraversive disruption than ipsiversive disruption” during saccade generation. The latter possibility is consistent with stronger ipsiversive RT delays, although the neuronal tuning in pre/perisaccadic epochs was weakly contralateral. The contraversive disruption assumption also does not account for contraversive evoked saccades unless the main role of pulvinar is to help maintaining fixation and ignore contralateral hemifield.

Yet another possibility is that stimulating neurons with RFs away from the target but within the same hemifield is more detrimental than when the RFs and the target are in opposite hemifields, thus leading to ipsiversive facilitation. However, the reasons for contraversive facilitation in later stimulation periods remain unexplained under this assumption.

### Functional implications and future directions

The effect on choices was present only in the visually-guided task, not the memory-guided task. Therefore, the choice bias is not driven by purely perceptual processing (otherwise, we would expect that the stimulation before or after visual cues affects subsequent choices) nor is it a purely motor consequence (otherwise, we should have seen effects before and after the Go signal). We suggest that the dPul contribution to the decision is crucial when the visuomotor contingencies have to be integrated rapidly and concomitantly with action selection. Alternatively, the pulvinar might affect the choices only when the target selection takes place in the presence of visual stimuli (which was not the case for memory-guided saccades); this conjecture needs to be tested in future experiments comparing memory-guided and visually-guided delayed saccades.

The interpretation of the alleged contraversive drive due to pulvinar stimulation is still open. In the simplest scenario, it could relate to attentional/behavioral saliency vector in the retinotopic reference frame. However, the spatial processing in the pulvinar, especially the dorsal part, might extend beyond purely visual aspects, contributing to gaze and postural encoding and perhaps to a prediction error (Grieve et al., 2000; Kanai et al., 2015). For example, the stimulation could affect the perceived direction of gaze relative to the head or the body or perceived body midline. Further experiments with manipulation of visuomotor and postural contingencies will address these possibilities. Another question is how general the biphasic choice effect is. VPul stimulation did not elicit an ipsiversive bias, but it would be interesting to test the same protocol in frontoparietal cortical areas interconnected with the dPul.

The inevitable conundrum of causal interference studies is to what extent the observed behavior depends on the functioning of the target area, as opposed to spread of in(activation) to neighboring structures and consequences of network effects. Site-specific patterns (Fig. 8) and their dissimilarity from patterns in adjacent SC and caudate suggest fairly localized effects, but we cannot exclude some current spread through intercalated thalamocortical fibers, brachium of SC, or neighboring PIp/m/cm subdivisions of inferior pulvinar (Stepniewska, 2004; Rosenberg et al., 2009). The pulvinar stimulation with a similar protocol during fMRI activates an extensive visuomotor cortical circuitry in the stimulated hemisphere, with distinct patterns for dorsal versus ventral sites consistent with anatomical connectivity (L. Gibson, M. Wilke, I. Kagan, unpublished observations). Therefore, the observed effects can be mediated by predominantly contralaterally tuned cortical areas (Kagan et al., 2010; Wilke et al., 2012). Future work combining epoch-specific stimulation with fMRI and electrophysiological readouts should elucidate the neuronal basis of these effects.

## References

[B1] Andersen RA, Cui H (2009) Intention, action planning, and decision making in parietal-frontal circuits. Neuron 63:568–583. 10.1016/j.neuron.2009.08.028 19755101

[B2] Arend I, Rafal R, Ward R (2008) Spatial and temporal deficits are regionally dissociable in patients with pulvinar lesions. Brain 131:2140–2152. 10.1093/brain/awn135 18669494

[B3] Bakker R, Tiesinga P, Kötter R (2015) The scalable brain atlas: instant web-based access to public brain atlases and related content. Neuroinformatics 13:353–366. 10.1007/s12021-014-9258-x 25682754 PMC4469098

[B4] Bender DB, Baizer JS (1990) Saccadic eye movements following kainic acid lesions of the pulvinar in monkeys. Exp Brain Res 79:467–478. 2340867 10.1007/BF00229317

[B5] Bender DB, Butter CM (1987) Comparison of the effects of superior colliculus and pulvinar lesions on visual search and tachistoscopic pattern discrimination in monkeys. Exp Brain Res 69:140–154. 3436384 10.1007/BF00247037

[B6] Bender DB, Youakim M (2001) Effect of attentive fixation in macaque thalamus and cortex. J Neurophysiol 85:219–234. 11152722 10.1152/jn.2001.85.1.219

[B7] Benevento LA, Port JD (1995) Single neurons with both form/color differential responses and saccade-related responses in the nonretinotopic pulvinar of the behaving macaque monkey. Vis Neurosci 12:523–544. 10.1017/S0952523800008439 7654609

[B8] Berman RA, Wurtz RH (2011) Signals conveyed in the pulvinar pathway from superior colliculus to cortical area MT. J Neurosci 31:373–384. 10.1523/JNEUROSCI.4738-10.2011 21228149 PMC6623455

[B9] Brainard DH (1997) The Psychophysics Toolbox. Spat Vis 10:433–436. 10.1163/156856897X00357 9176952

[B10] Calabrese E, Badea A, Coe CL, Lubach GR, Shi Y, Styner MA, Johnson GA (2015) A diffusion tensor MRI atlas of the postmortem rhesus macaque brain. Neuroimage 117:408–416. 10.1016/j.neuroimage.2015.05.072 26037056 PMC4512905

[B11] Carello CD, Krauzlis RJ (2004) Manipulating intent: evidence for a causal role of the superior colliculus in target selection. Neuron 43:575–583. 10.1016/j.neuron.2004.07.026 15312655

[B12] Cheney PD, Griffin DM, Van Acker GM 3rd (2013) Neural hijacking: action of high-frequency electrical stimulation on cortical circuits. Neuroscientist 19:434–441. 10.1177/1073858412458368 22968640 PMC3965182

[B13] Cicmil N., Cumming B.G., Parker A.J., and Krug K (2015) Reward modulates the effect of visual cortical microstimulation on perceptual decisions. eLife e07832. 26402458 10.7554/eLife.07832 PMC4616243

[B14] Clark KL, Armstrong KM, Moore T (2011) Probing neural circuitry and function with electrical microstimulation. Proc Biol Sci 278:1121–1130. 10.1098/rspb.2010.2211 21247952 PMC3049083

[B15] Crommelinck M, Roucoux A, Meulders M (1977) Eye movements evoked by stimulation of lateral posterior nucleus and puIvinar in the alert cat. Brain Res 124:361–366. 10.1016/0006-8993(77)90894-0 843953

[B16] Desmurget M, Song Z, Mottolese C, Sirigu A (2013) Re-establishing the merits of electrical brain stimulation. Trends Cogn Sci 17:442–449. 10.1016/j.tics.2013.07.002 23932195

[B17] Ding L, Gold JI (2012) Separate, causal roles of the caudate in saccadic choice and execution in a perceptual decision task. Neuron 75:865–874. 10.1016/j.neuron.2012.07.021 22958826 PMC3446771

[B18] Dorris MC, Klein RM, Everling S, Munoz DP (2002) Contribution of the primate superior colliculus to inhibition of return. J Cogn Neurosci 14:1256–1263. 10.1162/089892902760807249 12495530

[B19] Fetsch CR, Kiani R, Newsome WT, Shadlen MN (2014) Effects of cortical microstimulation on confidence in a perceptual decision. Neuron 83:797–804. 10.1016/j.neuron.2014.07.011 25123306 PMC4141901

[B20] Goldberg ME, Bushnell MC, Bruce CJ (1986) The effect of attentive fixation on eye movements evoked by electrical stimulation of the frontal eye fields. Exp Brain Res 61:579–584. 3956616 10.1007/BF00237584

[B21] Grieve KL, Acuña C, Cudeiro J (2000) The primate pulvinar nuclei: vision and action. Trends Neurosci 23:35–39. 10.1016/S0166-2236(99)01482-4 10631787

[B22] Gutierrez C, Cola MG, Seltzer B, Cusick C (2000) Neurochemical and connectional organization of the dorsal pulvinar complex in monkeys. J Comp Neurol 419:61–86. 10.1002/(SICI)1096-9861(20000327)419:1<61::AID-CNE4>3.0.CO;2-I 10717640

[B23] Hafed ZM, Krauzlis RJ (2012) Similarity of superior colliculus involvement in microsaccade and saccade generation. J Neurophysiol 107:1904–1916. 10.1152/jn.01125.2011 22236714 PMC3331665

[B24] Hafed ZM, Chen CY, Tian X (2015) Vision, perception, and attention through the lens of microsaccades: mechanisms and implications. Front Syst Neurosci 9:167. 10.3389/fnsys.2015.00167 26696842 PMC4667031

[B25] Hanks TD, Ditterich J, Shadlen MN (2006) Microstimulation of macaque area LIP affects decision-making in a motion discrimination task. Nat Neurosci 9:682–689. 10.1038/nn1683 16604069 PMC2770004

[B26] Histed MH, Ni AM, Maunsell JH (2013) Insights into cortical mechanisms of behavior from microstimulation experiments. Prog Neurobiol 103:115–130. 10.1016/j.pneurobio.2012.01.006 22307059 PMC3535686

[B27] Isoda M (2005) Context-dependent stimulation effects on saccade initiation in the presupplementary motor area of the monkey. J Neurophysiol 93:3016–3022. 10.1152/jn.01176.2004 15703225

[B28] Izawa Y, Suzuki H, Shinoda Y (2004a) Suppression of visually and memory-guided saccades induced by electrical stimulation of the monkey frontal eye field. II. Suppression of bilateral saccades. J Neurophysiol 92:2261–2273. 10.1152/jn.00085.2004 15381745

[B29] Izawa Y, Suzuki H, Shinoda Y (2004b) Suppression of visually and memory-guided saccades induced by electrical stimulation of the monkey frontal eye field. I. Suppression of ipsilateral saccades. J Neurophysiol 92:2248–2260. 10.1152/jn.01021.2003 15381744

[B30] Izawa Y, Suzuki H, Shinoda Y (2009) Response properties of fixation neurons and their location in the frontal eye field in the monkey. J Neurophysiol 102:2410–2422. 10.1152/jn.00234.2009 19675294

[B31] Jones EG (2012) The thalamus. New York: Springer.

[B32] Kaas JH, Lyon DC (2007) Pulvinar contributions to the dorsal and ventral streams of visual processing in primates. Brain Res Rev 55:285–296. 10.1016/j.brainresrev.2007.02.008 17433837 PMC2100380

[B33] Kagan I, Iyer A, Lindner A, Andersen RA (2010) Space representation for eye movements is more contralateral in monkeys than in humans. Proc Natl Acad Sci U S A 107:7933–7938. 10.1073/pnas.1002825107 20385808 PMC2867911

[B34] Kanai R, Komura Y, Shipp S, Friston K (2015) Cerebral hierarchies: predictive processing, precision and the pulvinar. Philos Trans R Soc Lond B Biol Sci 370: pii: 20140169. 10.1098/rstb.2014.0169 25823866 PMC4387510

[B35] Karnath HO, Himmelbach M, Rorden C (2002) The subcortical anatomy of human spatial neglect: putamen, caudate nucleus and pulvinar. Brain 125:350–360. 10.1093/brain/awf032 11844735

[B36] Komura Y, Nikkuni A, Hirashima N, Uetake T, Miyamoto A (2013) Responses of pulvinar neurons reflect a subject's confidence in visual categorization. Nat Neurosci 16:749–755. 10.1038/nn.3393 23666179

[B37] Lee C, Rohrer WH, Sparks DL (1988) Population coding of saccadic eye movements by neurons in the superior colliculus. Nature 332:357–360. 10.1038/332357a0 3352733

[B38] Logothetis NK, Augath M, Murayama Y, Rauch A, Sultan F, Goense J, Oeltermann A, Merkle H (2010) The effects of electrical microstimulation on cortical signal propagation. Nat Neurosci 13:1283–1291. 10.1038/nn.2631 20818384

[B39] Maldonado H, Joseph JP, Schlag J (1980) Types of eye movements evoked by thalamic microstimulation in the alert cat. Exp Neurol 70:613–625. 10.1016/0014-4886(80)90187-9 7439298

[B40] Mirpour K, Ong WS, Bisley JW (2010) Microstimulation of posterior parietal cortex biases the selection of eye movement goals during search. J Neurophysiol 104:3021–3028. 10.1152/jn.00397.2010 20861428 PMC3007667

[B41] Moeller S, Freiwald WA, Tsao DY (2008) Patches with links: a unified system for processing faces in the macaque temporal lobe. Science 320:1355–1359. 10.1126/science.1157436 18535247 PMC8344042

[B42] Munoz DP, Wurtz RH (1993a) Fixation cells in monkey superior colliculus. I. Characteristics of cell discharge. J Neurophysiol 70:559–575. 8410157 10.1152/jn.1993.70.2.559

[B43] Munoz DP, Wurtz RH (1993b) Fixation cells in monkey superior colliculus. II. Reversible activation and deactivation. J Neurophysiol 70:576–589. 8410158 10.1152/jn.1993.70.2.576

[B44] Murasugi CM, Salzman CD, Newsome WT (1993) Microstimulation in visual area MT: effects of varying pulse amplitude and frequency. J Neurosci 13:1719–1729. 8463847 10.1523/JNEUROSCI.13-04-01719.1993 PMC6576737

[B45] Murphey DK, Maunsell JH (2008) Electrical microstimulation thresholds for behavioral detection and saccades in monkey frontal eye fields. Proc Natl Acad Sci U S A 105:7315–7320. 10.1073/pnas.0710820105 18477698 PMC2438247

[B46] Ohayon S, Tsao DY (2012) MR-guided stereotactic navigation. J Neurosci Methods 204:389–397. 10.1016/j.jneumeth.2011.11.031 22192950 PMC8204678

[B47] Opris I, Barborica A, Ferrera VP (2005) Microstimulation of the dorsolateral prefrontal cortex biases saccade target selection. J Cogn Neurosci 17:893–904. 10.1162/0898929054021120 15969908

[B48] Petersen SE, Robinson DL, Keys W (1985) Pulvinar nuclei of the behaving rhesus monkey: visual responses and their modulation. J Neurophysiol 54:867–886. 4067625 10.1152/jn.1985.54.4.867

[B49] Preuss T.M (2007) Evolutionary specializations of primate brain systems. In: Primate origins: evolution and adaptations (Ravosa MJ, Dagosto M, eds). New York: Springer, p. 625–675.

[B50] Rafal RD, Posner MI (1987) Deficits in human visual spatial attention following thalamic lesions. Proc Natl Acad Sci U S A 84:7349–7353. 10.1073/pnas.84.20.7349 3478697 PMC299290

[B51] Rafal R, McGrath M, Machado L, Hindle J (2004) Effects of lesions of the human posterior thalamus on ocular fixation during voluntary and visually triggered saccades. J Neurol Neurosurg Psychiatry 75:1602–1606. 10.1136/jnnp.2003.017038 15489394 PMC1738817

[B52] Robinson DA, Fuchs AF (1969) Eye movements evoked by stimulation of frontal eye fields. J Neurophysiol 32:637–648. 4980022 10.1152/jn.1969.32.5.637

[B53] Robinson DL, Petersen SE (1992) The pulvinar and visual salience. Trends Neurosci 15:127–132. 10.1016/0166-2236(92)90354-B 1374970

[B54] Robinson DL, Petersen SE, Keys W (1986) Saccade-related and visual activities in the pulvinar nuclei of the behaving rhesus monkey. Exp Brain Res 62:625–634. 3720891 10.1007/BF00236042

[B55] Robinson DL, McClurkin JW, Kertzman C (1990) Orbital position and eye movement influences on visual responses in the pulvinar nuclei of the behaving macaque. Exp Brain Res 82:235–246. 2286229 10.1007/BF00231243

[B56] Rohlfing T, Kroenke CD, Sullivan EV, Dubach MF, Bowden DM, Grant KA, Pfefferbaum A (2012) The INIA19 template and NeuroMaps atlas for primate brain image parcellation and spatial normalization. Front Neuroinform 6:27. 10.3389/fninf.2012.00027 23230398 PMC3515865

[B57] Rosenberg DS, Mauguière F, Catenoix H, Faillenot I, Magnin M (2009) Reciprocal thalamocortical connectivity of the medial pulvinar: a depth stimulation and evoked potential study in human brain. Cereb Cortex 19:1462–1473. 10.1093/cercor/bhn185 18936272

[B58] Saalmann YB, Kastner S (2011) Cognitive and perceptual functions of the visual thalamus. Neuron 71:209–223. 10.1016/j.neuron.2011.06.027 21791281 PMC3148184

[B59] Saalmann YB, Pinsk MA, Wang L, Li X, Kastner S (2012) The pulvinar regulates information transmission between cortical areas based on attention demands. Science 337:753–756. 10.1126/science.1223082 22879517 PMC3714098

[B60] Scherberger H, Goodale MA, Andersen RA (2003) Target selection for reaching and saccades share a similar behavioral reference frame in the macaque. J Neurophysiol 89:1456–1466. 12612028 10.1152/jn.00883.2002

[B61] Schlag J, Schlag-Rey M, Dassonville P (1989) Interactions between natural and electrically evoked saccades. Exp Brain Res 76:548–558. 10.1007/BF00248911 2551712

[B62] Seltzer B, Cola MG, Gutierrez C, Massee M, Weldon C, Cusick CG (1996) Overlapping and nonoverlapping cortical projections to cortex of the superior temporal sulcus in the rhesus monkey: double anterograde tracer studies. J Comp Neurol 370:173–190. 10.1002/(SICI)1096-9861(19960624)370:2<173::AID-CNE4>3.0.CO;2-# 8808729

[B63] Shadlen MN, Kiani R (2013) Decision making as a window on cognition. Neuron 80:791–806. 10.1016/j.neuron.2013.10.047 24183028 PMC3852636

[B64] Sherman SM, Guillery RW (2002) The role of the thalamus in the flow of information to the cortex. Philos Trans R Soc Lond B Biol Sci 357:1695–1708. 10.1098/rstb.2002.1161 12626004 PMC1693087

[B65] Shibutani H, Sakata H, Hyvärinen J (1984) Saccade and blinking evoked by microstimulation of the posterior parietal association cortex of the monkey. Exp Brain Res 55:1–8. 6745342 10.1007/BF00240493

[B66] Snow JC, Allen HA, Rafal RD, Humphreys GW (2009) Impaired attentional selection following lesions to human pulvinar: evidence for homology between human and monkey. Proc Natl Acad Sci U S A 106:4054–4059. 10.1073/pnas.0810086106 19237580 PMC2656203

[B67] Stepniewska I (2004) The pulvinar complex. In: The primate visual system (Kaas JH, Collins CE, ed.), pp 53–80. Boca Raton, FL: CRC Press.

[B68] Tehovnik EJ, Slocum WM, Schiller PH (1999) Behavioural conditions affecting saccadic eye movements elicited electrically from the frontal lobes of primates. Eur J Neurosci 11:2431–2443. 10.1046/j.1460-9568.1999.00665.x 10383633

[B69] Tehovnik EJ, Slocum WM, Schiller PH (2002) Differential effects of laminar stimulation of V1 cortex on target selection by macaque monkeys. Eur J Neurosci 16:751–760. 10.1046/j.1460-9568.2002.02123.x 12270051

[B70] Thier P, Andersen RA (1996) Electrical microstimulation suggests two different forms of representation of head-centered space in the intraparietal sulcus of rhesus monkeys. Proc Natl Acad Sci U S A 93:4962–4967. 10.1073/pnas.93.10.4962 8643512 PMC39388

[B71] Van der Stigchel S, Arend I, van Koningsbruggen MG, Rafal RD (2010) Oculomotor integration in patients with a pulvinar lesion. Neuropsychologia 48:3497–3504. 10.1016/j.neuropsychologia.2010.07.035 20691714

[B72] Vanni MP, Thomas S, Petry HM, Bickford ME, Casanova C (2015) Spatiotemporal profile of voltage-sensitive dye responses in the visual cortex of tree shrews evoked by electric microstimulation of the dorsal lateral geniculate and pulvinar nuclei. J Neurosci 35:11891–11896. 10.1523/JNEUROSCI.0717-15.2015 26311771 PMC4549400

[B73] Watanabe M, Munoz DP (2010) Saccade suppression by electrical microstimulation in monkey caudate nucleus. J Neurosci 30:2700–2709. 10.1523/JNEUROSCI.5011-09.2010 20164354 PMC6634530

[B74] Watanabe M, Munoz DP (2011) Saccade reaction times are influenced by caudate microstimulation following and prior to visual stimulus appearance. J Cogn Neurosci 23:1794–1807. 10.1162/jocn.2010.21554 20666599

[B75] Watanabe M, Munoz DP (2013) Effects of caudate microstimulation on spontaneous and purposive saccades. J Neurophysiol 110:334–343. 10.1152/jn.00046.2013 23636720

[B76] Wegener SP, Johnston K, Everling S (2008) Microstimulation of monkey dorsolateral prefrontal cortex impairs antisaccade performance. Exp Brain Res 190:463–473. 10.1007/s00221-008-1488-4 18641976

[B77] Wilke M, Turchi J, Smith K, Mishkin M, Leopold DA (2010) Pulvinar inactivation disrupts selection of movement plans. J Neurosci 30:8650–8659. 10.1523/JNEUROSCI.0953-10.2010 20573910 PMC2905633

[B78] Wilke M, Kagan I, Andersen RA (2012) Functional imaging reveals rapid reorganization of cortical activity after parietal inactivation in monkeys. Proc Natl Acad Sci U S A 109:8274–8279. 10.1073/pnas.1204789109 22562793 PMC3361455

[B79] Wilke M, Kagan I, Andersen RA (2013) Effects of pulvinar inactivation on spatial decision-making between equal and asymmetric reward options. J Cogn Neurosci 25:1270–1283. 10.1162/jocn_a_00399 23574581

[B80] Yamamoto S, Monosov IE, Yasuda M, Hikosaka O (2012) What and where information in the caudate tail guides saccades to visual objects. J Neurosci 32:11005–11016. 10.1523/JNEUROSCI.0828-12.2012 22875934 PMC3465728

[B81] Yang SN, Heinen SJ, Missal M (2008) The effects of microstimulation of the dorsomedial frontal cortex on saccade latency. J Neurophysiol 99:1857–1870. 10.1152/jn.00119.2007 18216220

[B82] Zhou H, Schafer RJ, Desimone R (2016) Pulvinar-cortex interactions in vision and attention. Neuron 89:209–220. 10.1016/j.neuron.2015.11.034 26748092 PMC4723640

